# Influenza virus infection reprograms cholesterol biosynthesis to facilitate virus replication by the TAK1-RORγ axis

**DOI:** 10.1371/journal.ppat.1013646

**Published:** 2025-10-24

**Authors:** Jingting Zhang, Ruixuan Cao, Yujie Wang, Yuling Sun, Xiaoyue Ji, Penggang Liu, Kaituo Liu, Jing Sun, Xiaojun Chen, Demin Cai, Pinghu Zhang, Xiaoquan Wang, Xiufan Liu, Xiulong Xu

**Affiliations:** 1 College of Veterinary Medicine, Institute of Comparative Medicine, Yangzhou University, Yangzhou, China; 2 Joint International Research Laboratory of Agriculture and Agri-Product Safety of Ministry of Education of China, Yangzhou University, Yangzhou, China; 3 Jiangsu Key Laboratory of Pathogen Biology, Department of Pathogen Biology and Immunology, School of Basic Medical Sciences, Nanjing Medical University, Nanjing, China; 4 College of Animal Science and Technology, Yangzhou University, Yangzhou, China; 5 College of Medicine, Yangzhou University, Yangzhou, China; 6 Animal Infectious Disease Laboratory, College of Veterinary Medicine, China; 7 Jiangsu Co-innovation Center for Prevention and Control of Important Animal Infectious Diseases and Zoonosis, Yangzhou University, Yangzhou, China; Dalhousie University, CANADA

## Abstract

Infection and replication of enveloped viruses require host cells to supply substantial amounts of cellular cholesterol for processes such as binding, entry, trafficking, assembly, and budding. However, the mechanisms by which influenza A virus (IAV) regulates cholesterol biosynthesis remain poorly understood. In this study, we demonstrate that IAV infection induces the expression of the retinoic acid-related orphan receptor γ (RORγ), an orphan nuclear receptor, which cooperates with the sterol regulatory element-binding protein-2 (SREBP2) to regulate the expression of the 3-hydroxy-3-methylglutaryl coenzyme-A (HMG-CoA) reductase (HMGCR), a key enzyme in cholesterol biosynthesis. RORγ knockout and treatment with two RORγ inhibitors, XY018 and GSK805, suppress IAV-induced HMGCR expression, cholesterol biosynthesis, and viral replication. Notably, exogenous cholesterol rescues the inhibitory effect of XY018 on viral replication. Mechanistically, we show that IAV infection activates RORγ expression through the TGF-β-activated kinase 1 (TAK1) and its downstream kinases, the c-Jun N-terminal kinase (JNK) and the IκB kinase (IKK), which in turn activate AP1 and NF-κB. In vivo, RORγ knockout reduces IAV replication, alleviates body weight loss, and prolongs survival in infected mice. Furthermore, XY018 treatment reduces both viral replication and inflammation in the lungs of IAV-infected mice. Our findings provide novel mechanistic insights into how IAV infection upregulates cholesterol biosynthesis to facilitate viral replication.

## Author summary

Conventional anti-cholesterol drugs targeting HMGCR have limited antiviral activity due to their poor ability to lower intracellular cholesterol levels. Understanding how viruses regulate cholesterol synthesis in infected cells is crucial for developing more effective therapies. In this study, we demonstrate that influenza A virus (IAV) induces the expression of RORγ, a key transcription factor involved in cholesterol biosynthesis, through activation of TAK1. Inhibition of RORγ, either by specific inhibitors or gene knockout, effectively suppresses IAV replication both in vitro and in vivo. Our findings offer new mechanistic insights into the regulation of cholesterol biosynthesis during viral infection and suggest that targeting RORγ could provide a promising strategy for developing novel antiviral therapies against IAV and potentially other emerging viral threats.

## Introduction

Influenza is a highly contagious respiratory disease that often causes seasonal epidemics and significant morbidity and mortality worldwide [1]. Since the 2009 flu pandemic, the strains that emerged during that period continue to cause severe illness and high mortality rates, particularly among young adults and children. Influenza A virus (IAV) infects a wide range of avian and mammalian hosts, with recent reports highlighting the transmission of the H5N1 clade 2.3.4.4b avian influenza virus into dairy cattle, where it replicates efficiently in mammary glands and is released into cow milk, presenting an additional pandemic risk [2–5]. Vaccination remains the primary method of influenza prevention [6]. However, the frequent mutations and reassortment events in newly circulating IAV strains pose significant challenges to the development of highly effective vaccines. Antiviral drugs targeting neuraminidase and the M2 ion channel often demonstrate limited efficacy against newly emerged IAV strains [7,8]. In response, the past decade has seen increased efforts to target cellular factors as an alternative strategy for novel antiviral development [9]. For instance, Janus kinases (JAK), mTOR, and dihydroorotate dehydrogenase (DHO-DHase), a key enzyme in pyrimidine nucleotide synthesis, have all been implicated in IAV replication [9]. Although inhibitors of these enzymes show antiviral activity in vitro, many lack robust efficacy in vivo [9]. Thus, identifying new cellular factors crucial for IAV replication—and amenable to drug targeting—is essential for advancing antiviral therapies.

Cholesterol, a vital component of cellular membranes and organelles, plays a key role in virus binding, entry, trafficking, assembly, and budding. Intermediate products in cholesterol biosynthesis are also involved in protein modification and other critical biological processes. SREBP2, a transcription factor that regulates cholesterol synthesis, is activated when intracellular cholesterol levels are low. Under these conditions, SREBP2 moves from the endoplasmic reticulum to the Golgi, where it is cleaved and the N-terminal domain (nSREBP2) translocates to the nucleus to upregulate the expression of cholesterol biosynthesis-related genes, including HMGCR, a rate-limiting enzyme [10,11]. HMGCR, the target of statins, plays a central role in cholesterol biosynthesis. Its stability is regulated by RNF145- and gp78-mediated ubiquitination, while its activity is suppressed by AMP-activated kinase (AMPK)-mediated phosphorylation [12].

Emerging evidence suggests that lipid metabolism is reprogrammed during viral infection, enhancing both viral replication and inflammation. A recent study found that oleoyl-acyl-carrier-protein (ACP) hydrolase, an enzyme involved in lipid droplet production, is linked to virus replication and inflammation severity in IAV infections [13]. Cholesterol is critical for the entry, trafficking, and budding of enveloped viruses such as influenza, Zika, Ebola, and coronaviruses [10,14]. High-cholesterol diets in mice result in hyperlipidemia and increased susceptibility to IAV infection [15], while IAV replicates more efficiently in apolipoprotein E-deficient mice, exacerbating lung pathology [16]. Targeting cholesterol biosynthesis has thus emerged as a promising strategy for antiviral therapy. Statin therapy has been shown to reduce mortality in patients hospitalized with both common and severe pneumonia [17,18]. However, a study by Kwong et al. [19] reported no significant protective effect of statins in pneumonia patients over 10 influenza seasons in Ontario, Canada. Traditional cholesterol-lowering drugs such as statins activate SREBP2 in a feedback manner, leading to the upregulation of cholesterol synthesis and low-density lipoprotein (LDL) receptor expression, which may limit their efficacy in reducing viral replication [20,21]. These limitations underscore the need for a deeper understanding of how viruses regulate cholesterol biosynthesis and the identification of more effective molecular targets.

RORγ is an orphan nuclear receptor that regulates several critical cellular functions including inflammation and lipid metabolism [22]. RORγ deletion results in reduced expression of cholesterol biosynthesis genes, lower body weight in mice, and a significantly reduced risk of diabetes and insulin resistance [23,24]. Elevated RORγ expression has been associated with triple-negative breast cancer and acute leukemia [25,26]. Inhibitors of RORγ, such as XY018 and GSK805, have been shown to inhibit tumor growth in these cancers [25,26]. In this study, we investigate the role of RORγ in regulating cholesterol biosynthesis and virus replication. We demonstrate that IAV infection induces RORγ expression, which enhances cholesterol biosynthesis and virus replication. Mechanistically, IAV activates TAK1 and its downstream kinases, IKK and JNK, which in turn activate NF-κB and AP1 to induce RORγ expression. Our findings uncover a previously unrecognized role for RORγ in regulating cholesterol biosynthesis and viral replication, providing new insights into potential therapeutic strategies for controlling IAV infection.

## Results

**Fig 1:**
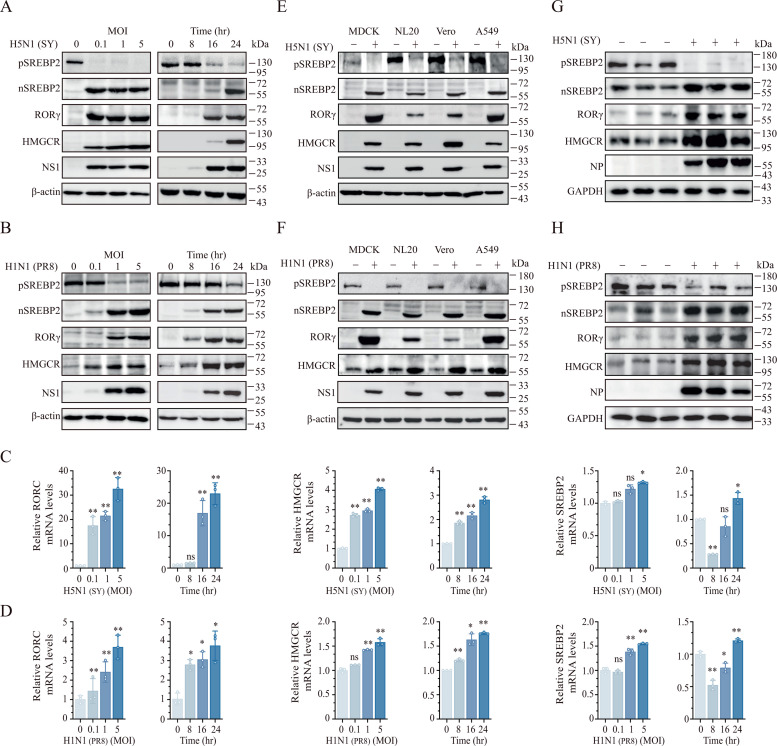
IAV induces the expression of cholesterol biosynthesis-related genes in vitro and in vivo. (A-D) NL20 were infected with various MOIs of H5N1 (SY) or H1N1 (PR8) viruses for 24 h or infected with 0.1 MOI of H5N1 or H1N1 virus for the indicated length of time. Cell lysates were prepared and analyzed for the levels of the indicated proteins by Western blot (A and B). The results represent one of three independent experiments with similar results. (C and D) Total RNAs were extracted and analyzed for the mRNA levels of cholesterol biosynthesis-related genes by RT-qPCR. Data are the means ± standard deviation (SD) of three independent experiments. ns, non-significant; *p < 0.05, **p < 0.01, compared to uninfected controls. (E and F) MDCK, NL20, Vero, and A549 cells were left uninfected or infected with 1 MOI of H5N1 (SY) or H1N1 (PR8) virus for 24 h. Cell lysates were prepared and analyzed for the levels of the indicated proteins by Western blot. (G and H) Male C57BL/6 mice (6-8-week-old, 3 mice/group) were intranasally infected with H5N1 (5 × 10^4^ pfu/mouse) or H1N1 (100 pfu/mouse) for 3 days, the lungs were collected and homogenized in the radioimmunoprecipitation assay buffer (RIPA) buffer and then analyzed for the levels of cholesterol biosynthesis-related proteins by Western blot. GAPDH was detected as a loading control.

**IAV Infection Induces ROR**γ **Expression and Activates SREBP2.** We first investigated the effect of IAV infection on RORγ expression and SREBP2 activation. Infection with H5N1 (SY) and H1N1 (PR8) viruses induced RORγ and HMGCR expression in a dose- and time-dependent manner, along with an increase in the active form of nuclear SREBP2 (nSREBP2) and a decrease in the full-length SREBP2 precursor (pSREBP2) in NL20 cells, a human bronchial noncancerous epithelial cell line (Fig 1A and 1B). Notably, the HMGCR protein induced by PR8 infection was observed as a processed 55-kDa form, consistent with findings reported by others [27,28]. Similar results were obtained in LET1 cells (a murine noncancerous alveolar epithelial cell line) and 293T cells (a human kidney cell line) (S1A and S1B Fig). UV-inactivated IAV failed to induce RORγ or HMGCR expression or activate SREBP2 in NL20 cells (S1C Fig), suggesting that virus replication is necessary for enhancing cholesterol biosynthesis. Consistent with this, H5N1 and H1N1 infections significantly increased the levels of RORγ (RORC) and HMGCR mRNAs but only modestly or weakly upregulated SREBP2 mRNA levels in NL20 cells (Fig 1C and 1D). H5N1 and H1N1 infections also induced RORγ and HMGCR expression and activated SREBP2 in three other mammalian cell lines: MDCK, Vero, and A549 (Fig 1E and 1F). In vivo, the levels of RORγ, HMGCR, and nSREBP2 proteins were significantly higher in the lungs of C57BL/6 mice infected with H5N1 or H1N1 for 3 days compared to mock-infected controls (Fig 1G and 1H). In contrast, pSREBP2 levels were reduced in the lungs of virus-infected mice (Fig 1G and 1H). Collectively, these findings suggest that IAV infection strongly induces RORγ and HMGCR expression and activates SREBP2 both in vitro and in vivo.

**Fig 2:**
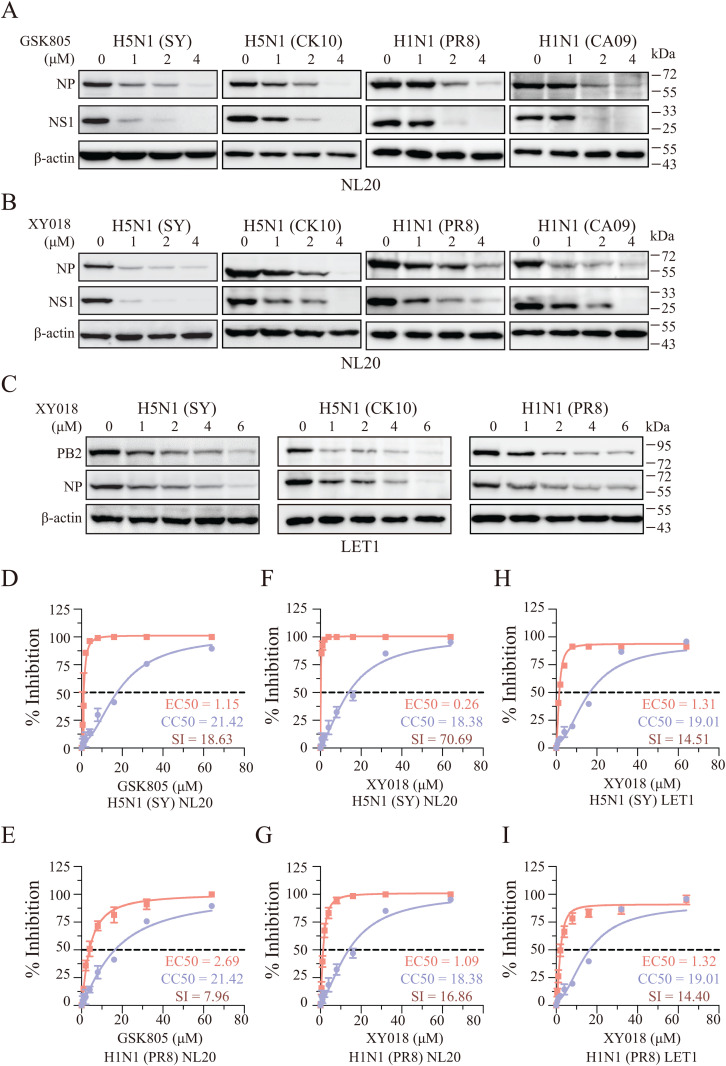
RORγ inhibitors inhibit IAV replication. (A and B) NL20 cells pretreated with the indicated concentrations of GSK805 (**A**) or XY018 (**B**) for 8 h were infected with 0.01 MOI of two H5N1 strains (SY & CK10) or 1 MOI of two H1N1 strains (PR8 & CA09) and then incubated for 24 h in the presence of the same concentrations of GSK805 or XY018. (C) LET1 cells pretreated with the indicated concentrations of XY018 for 8 h were infected with 0.01 MOI of two H5N1 strains (SY & CK10) or 1 MOI of the H1N1 virus (PR8) and then incubated for 24 h in the presence of the same concentrations of XY018. Untreated control cells were treated with 0.1% dimethyl sulfoxide (DMSO). Cell lysates were prepared and analyzed for the levels of the indicated proteins by Western blot. β-actin was detected as a loading control. (**D-I**) NL20 and LET1 cells seeded in a 96-well plate (3.5 × 10^4^ cells/well) were incubated in the absence or presence of the indicated concentrations of GSK805 or XY018 in triplicate for 48 h. Cell viability was measured by using the CellTiter-Glo kit. The CC_50_ values were calculated based on the mean ± SD of three experiments. To determine the EC_50_ values, NL20 and LET1 cells seeded in a 24-well plate were pretreated with the indicated concentrations of GSK805 or XY018 for 8 h. After infection with 0.01 MOI H5N1 (SY) or 1 MOI of the H1N1 (PR8) virus, the cells were incubated in the absence or presence of the same concentrations of GSK805 or XY018 for 24 h. The conditioned media were collected and analyzed for virus titers by measuring TCID_50_ values. The results represent the mean ± SD of three independent experiments. The selective index (S.I.) values were calculated by dividing the CC_50_ values with the EC_50_ values.

**Inhibition of IAV Replication by RORγ Inhibitors**. Next, we tested whether GSK805 and XY018, two RORγ-specific inhibitors [25,26], could suppress IAV replication. Both GSK805 and XY018 dose-dependently reduced the levels of viral NS1 and NP proteins in NL20 cells infected with two H5N1 strains (SY & CK10) and two H1N1 strains (PR8 & CA09) (Fig 2A and 2B). XY018 also decreased the levels of viral PB2 and NP proteins in LET1 cells infected with H5N1 (SY & CK10) and H1N1 (PR8) strains (Fig 2C). Additionally, both GSK805 and XY018 lowered virus titers in the conditioned media of H5N1 (SY) or H1N1 (PR8)-infected NL20 cells (Fig 2D-G). The half maximal effective concentration (EC_50_) values of GSK805 to inhibit H5N1 and H1N1 replication in NL20 cells were 1.15 μM and 2.69 μM, respectively (Fig 2D and 2E). Similarly, XY018 had EC_50_ values of 0.26 μM and 1.09 μM for H5N1 and H1N1 virus replication in NL20 cells, respectively (Fig 2F and 2G). Both inhibitors showed minimal cytotoxicity, with 50% cytotoxic concentration (CC_50_) values of 21.42 μM for GSK805 and 18.38 μM for XY018 (Fig 2D-G). The selective index (S.I.) values for GSK805 against H5N1 and H1N1, which is calculated by dividing the CC_50_ value with the EC_50_ value, were 18.63 and 7.96, respectively (Fig 2D and 2E), while the S.I. values for XY018 were 70.69 and 16.86, respectively (Fig 2F and 2G). For LET1 cells, the EC_50_ values of XY018 against H5N1 and H1N1 were 1.31 μM and 1.32 μM, respectively (Fig 2H and 2I), and the CC_50_ value was 19.01 μM (Fig 2H and 2I), with corresponding S.I. values of 14.51 and 14.40 (Fig 2H and 2I). The S.I. values of XY018 and GSK805 in NL20 and LET1 cells infected with both subtypes are near or bigger than 10, suggesting that both compounds exert their antiviral effect not by their cytotoxic effect. Notably, H5N1 appears to be more sensitive to RORγ inhibitors than H1N1, which may be due to its faster replication rate and the earlier depletion of intracellular cholesterol. Additionally, H5N1 activates JNK more readily than H1N1 (PR8) [29,30], which could further explain the observed differences.

**Fig 3:**
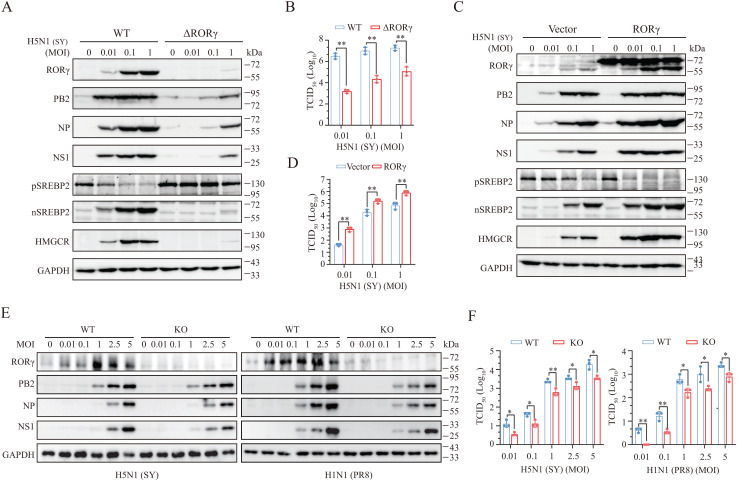
RORγ knockout inhibits IAV replication. (A and B) Control and RORγ-deficient 293T cells were infected with the indicated MOIs of H5N1 (SY) and then incubated for 16 h. Cell lysates were prepared and analyzed for the expression of indicated proteins (A). Conditioned media were collected for measuring TCID_50_ values (B). WT, wild-type; △RORγ, RORγ deficiency. (C and D) 293T cells were transfected with the empty vector or the vector encoding RORγ. After incubation for 48 h, cells were infected with the indicated MOIs of H5N1 (SY) virus and incubated for an additional 16 h. Cell lysates were prepared and analyzed for cholesterol biosynthesis-related proteins and viral proteins levels (C). Conditioned media were collected for measuring TCID_50_ values (D). (E and F) WT and RORγ-deficient MEF cells were infected with the indicated MOIs of H5N1 (SY) or H1N1 (PR8) and then incubated for 12 h. Cell lysates were prepared and analyzed for viral proteins levels. Virus titers in the conditioned media were analyzed by measuring TCID_50_ values. The data in **B, D**, and **F** represent the mean ± SD of three independent experiments. *p < 0.05; **p < 0.01.

**RORγ Promotes IAV Replication**. RORγ knockout in 293T cells lowered the levels of PB2, NP, and NS1, HMGCR, and nSREBP2 (Fig 3A) and virus titers in the conditioned media (Fig 3B). Similar results were observed in RORγ knockdown NL20 cells (S2A Fig). RORγ overexpression in 293T cells increased the levels of PB2, NP, NS1, HMGCR, and nSREBP2 and virus titers (Fig 3C and 3D). Similar observations were made in NL20 cells overexpressing RORγ (S2B Fig). Consistent with these findings, RORγ deficiency lowered the levels of PB2, NP, and NS1 proteins and decreased virus titers in murine embryonic fibroblasts (MEFs) infected with H5N1 and H1N1 viruses (Fig 3E and 3F). IAV infection also induced RORγ expression in wild-type MEFs (Fig 3E). These results collectively suggest that RORγ plays a crucial role in IAV replication, and that the role of RORγ in IAV replication is not cell-type specific.

**Fig 4:**
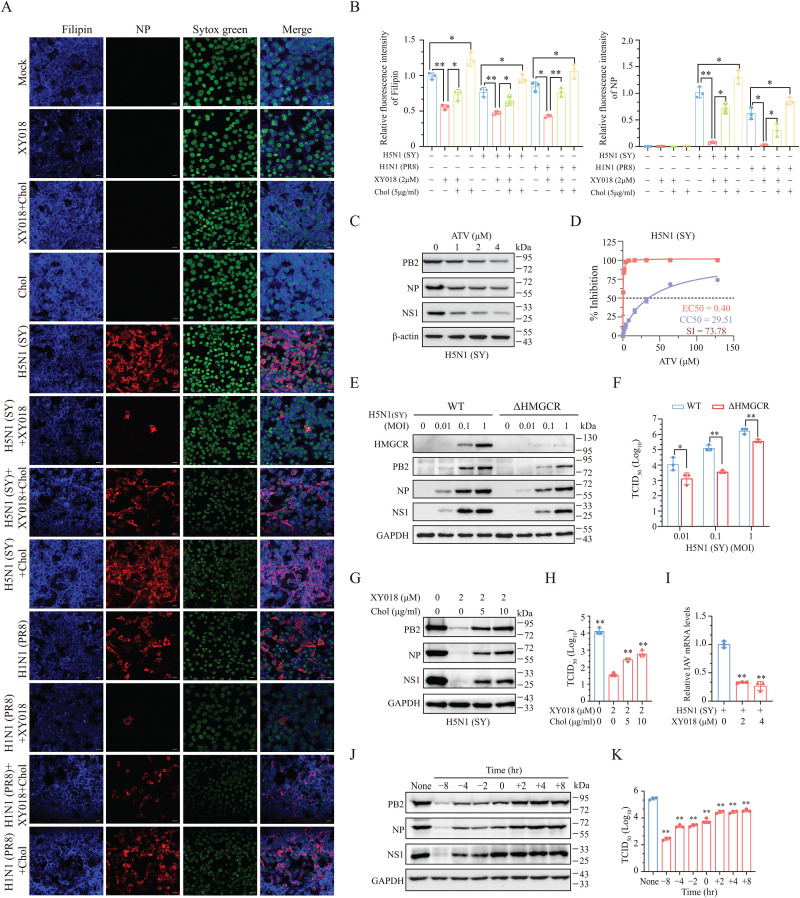
RORγ enhances IAV replication by enhancing cholesterol biosynthesis. (A) The monolayers of NL20 cells seeded on coverslips were pre-incubated in the absence of presence of XY018 (2 μM) or cholesterol (5 μg/ml) for 4 h and then infected with H5N1 (SY) (1 MOI) or H1N1 (PR8) (5 MOI) virus. After incubation in serum-free media for 16 h, cells were stained for cholesterol with Filipin and for nuclei with Sytox Green as an internal control, and for NP with a monoclonal antibody. Cells were visualized and photographed under a fluorescent microscope. Scale bar, 10 μm. (B) The monolayers of NL20 cells grown in a 96-well plate were pre-incubated in the absence or presence of XY018 (2 μM) or cholesterol (5 μg/ml) for 4 h and then infected with H5N1 (SY) (1 MOI) or H1N1 (PR8) (5 MOI) virus. After incubation in serum-free media for 16 h, cells were stained for cholesterol with Filipin and for nuclei with Sytox Green as an internal control, and for NP with a monoclonal antibody. The plate was read in a microplate reader for the fluorescent signals of Filipin and Sytox Green. The arbitrary units of Filipin fluorescence intensity were normalized with that of Sytox Green intensity. The results represent the mean ± SD of three independent experiments. *p < 0.05; **p < 0.01. (C) NL20 cells pretreated with the indicated concentrations of Atovastatin (ATV) for 8 h were infected with 0.01 MOI H5N1 (SY) and then incubated for 24 h in the presence of the same concentrations of ATV. Untreated control cells were treated with 0.1% DMSO. Cell lysates were prepared and analyzed for the levels of indicated proteins by Western blot. β-actin was detected as a loading control. (D) NL20 cells seeded in a 96-well plate (3.5 × 10^4^ cells/well) were incubated in the absence or presence of the indicated concentrations of ATV in triplicate for 48 h. Cell viability was measured by using the CellTiter-Glo kit. The CC_50_ values were calculated based on the mean ± SD of three independent experiments. To determine the EC_50_ values, NL20 cells seeded in a 24-well plate were pretreated with the indicated concentrations of ATV for 8 h. After infection with 0.01 MOI H5N1 (SY) virus, the cells were incubated in the absence or presence of the same concentrations of ATV for 24 h. The conditioned media were collected and analyzed for virus titers by measuring TCID_50_ values. The results represent the mean ± SD of three independent experiments. The S.I. values were calculated by dividing the CC_50_ values with the EC_50_ values. (E and F) Wild-type control and HMGCR-deficient (△HMGCR) NL20 cells infected with the indicated MOIs of H5N1 (SY) virus were incubated for 16 h. Cell lysates were analyzed for the expression of viral proteins. The results represent one of three independent experiments with similar results. Conditioned medial were collected and analyzed for virus titers by measuring TCID_50_ values (F). Data are the mean ± SD of three independent experiments. **p < 0.01. (G and H) NL20 cells pre-incubated in the absence or presence of cholesterol (5 or 10 μg/ml) minus or plus XY018 (2 μM) for 8 h were infected with 0.01 MOI of H5N1 (SY) virus and incubated for 24 h. Cell lysates were prepared and analyzed for the PB2, NP, and NS1 proteins. (H) Conditioned media were collected and analyzed for virus titers. Data are the mean ± SD of three independent experiments. **p < 0.01. (I) NL20 cells pretreated in serum-free media containing XY018 (2 μM) for 8 h were infected with 2.5 MOI H5N1 (SY) virus and incubated at 4 °C for 1 h. After removing unattached viruses, total cellular RNAs were immediately extracted and analyzed for viral RNAs by RT-qPCR. Data are the mean ± SD of three independent experiments. **p < 0.01. (J and K) NL20 cells were treated with XY018 (2 μM) for the indicated timepoints before or after H5N1 (SY) virus infection (0.01 MOI). Cell lysates were prepared and analyzed for viral proteins (J). Conditioned media were collected 24 h post infection and analyzed for virus titers (K). Data are the mean ± SD of three experiments. **p < 0.01, compared to the untreated control.

**RORγ Enhances IAV Replication by Upregulating Cholesterol Biosynthesis.** We first tested if inhibition of RORγ activity impacted cellular cholesterol levels. XY018 significantly reduced intracellular cholesterol content in uninfected NL20 cells (Fig 4A and 4B) and lowered the levels of both cholesterol and the NP protein in H5N1 or H1N1 virus-infected NL20 cells. Although IAV infection induced RORγ expression and activated SREBP2, both H5N1 and H1N1 viruses decreased cellular cholesterol levels in NL20 cells (Fig 4A and 4B). This is likely due to cholesterol depletion during virion assembly and budding. Notably, H1N1 virus infection had a lesser impact on intracellular cholesterol levels than H5N1, probably due to its slower replication rate (Fig 4A and 4B). Supplementation with exogenous cholesterol increased the levels of intracellular cholesterol in uninfected NL20 cells and restored the levels of intracellular cholesterol in IAV-infected or XY018-treated cells (Fig 4A and 4B).

HMGCR is a key enzyme in cholesterol biosynthesis and is transcriptionally regulated by RORγ. The HMGCR inhibitor atorvastatin (ATV) dose-dependently reduced the levels of viral PB2, NP, and NS1 proteins (Fig 4C). The EC_50_ of ATV to inhibit H5N1 virus replication in NL20 cells was 0.40 μM (Fig 4D), with a CC_50_ value of 29.51 μM (Fig 4D) and a S.I. value of 73.78 (Fig 4D). HMGCR knockout in NL20 cells also significantly decreased viral PB2, NP, and NS1 proteins (Fig 4E) and reduced virus titers (Fig 4F). HMGCR knockout suppresses viral replication, providing further support for the idea that RORγ promotes IAV replication by inducing HMGCR expression. Moreover, adding exogenous cholesterol to the cell culture partially restored the levels of these viral proteins and virus titers in H5N1-infected NL20 cells treated with XY018 (Fig 4G and 4H). These data suggest that XY018 inhibits IAV replication by inhibiting RORγ activity and lowering cellular cholesterol levels.

Cholesterol in lipid rafts plays a key role in IAV endocytosis [31–34]. IAV co-localizes with host membrane lipid rafts during binding, and lipid depletion by methyl-β-cyclodextrin reduces IAV binding to MDCK cells, suggesting that cholesterol is necessary for IAV binding [35]. Here, we tested whether XY018-induced cholesterol depletion in NL20 cells would affect virus binding. XY018 pretreatment decreased virus binding to the cell surface (Fig 4I). To confirm the role of cellular cholesterol in virus binding, NL20 cells pretreated with XY018 for varying time points before or after infection were inoculated with H5N1 virus at a multiplicity of infection (MOI) of 0.01. Pretreatment for 8 hours strongly inhibited virus replication (Fig 4J and 4K). However, adding XY018 2 hours or later post-infection had a modest or negligible effect on viral protein levels and virus titers in the conditioned media (Fig 4J and 4K). These findings suggest that XY018 interferes with virus infection in part by blocking virus binding.

**Fig 5:**
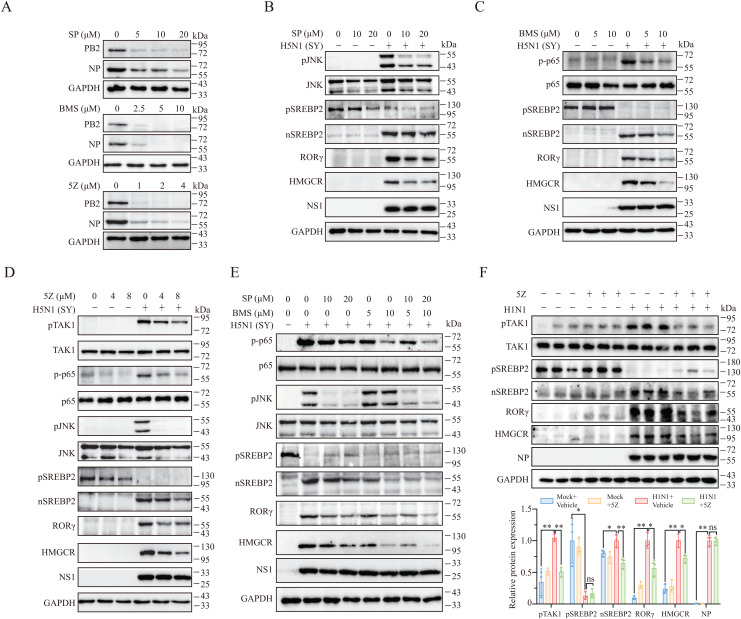
TAK1, JNK, and IKK inhibitors suppress IAV replication and RORγ expression. (A) NL20 cells were infected with 0.1 MOI of H5N1 (SY) virus and then treated with the indicated concentrations of the JNK inhibitor (SP600125, SP), the IKK inhibitor (BMS345541. BMS) and the TAK1 inhibitor (5Z-oxzeneonal, 5Z) for 24 h. Cell lysates were prepared and analyzed for the levels of the PB2 and NP proteins by Western blots. GAPDH was detected as a loading control. (B-E) NL20 cells were first infected with 0.1 MOI of H5N1 virus (SY). After incubation for 8 h, the cells were then incubated in the absence or presence of the indicated concentrations of SP, BMS, or 5Z alone (**B-D**) or SP plus BMS **(E)** for 16 h. Cell lysates were prepared and analyzed for the levels of the indicated proteins by Western blot. GAPDH was detected as a loading control. The experiments were repeated three times with similar results. (F) Female C57BL/6 mice were randomly divided into 4 groups (3 mice/group). The mice were either mock-infected or infected with H1N1 virus (1000 pfu/mouse). One day after infection, the mice were treated daily with the vehicle or 5Z at a dose of 2 mg/kg bodyweight for two consecutive days. On the third day post-infection, the mice received a last dose of 5Z at 8 hours prior to sacrifice. Lung tissue lysates were prepared and analyzed for the levels of the indicated proteins by Western blot. The band densities from 3 mice per group were analyzed using NIH Image-J software and normalized by the arbitrary units of their total protein bands or GAPDH levels. ns, non-significant, ^*^p < 0.05; ^**^p < 0.01.

**TAK1 Activates JNK and IKK to Induce RORγ Expression and Enhance Virus Replication.** We next explored the mechanisms underlying IAV-induced RORγ expression. Previous studies have identified AP1 and NF-κB transcription factor binding sites in the RORγ (*RORC*) gene [36,37]. IAV RNA binds the Toll-like receptor 3 (TLR3), activating TAK1 and its downstream kinases JNK and IKK, which in turn activate AP1 and NF-κB, respectively [38]. We hypothesized that IAV infection induces RORγ expression by activating TAK1 and its downstream signaling pathways. The inhibitors of JNK (SP600125, SP), IKK (BMS-345541, BMS), and TAK1 (5Z-7-oxozeaenol, 5Z) all suppressed virus replication, as indicated by reduced levels of PB2 and NP proteins (Fig 5A). To confirm that inhibition of RORγ expression by these inhibitors were not due to inhibition of virus replication by other mechanism of action, the inhibitors were added 8 hr post virus infection. Indeed, these inhibitors did not affect the NS1 levels but effectively blocked JNK, p65, and TAK1 phosphorylation (Fig 5B-D). However, these inhibitors did modestly inhibit RORγ expression at low and high concentration (Fig 5B-D). Co-inhibition of JNK and IKK synergistically suppressed RORγ and HMGCR expression and SREBP2 activation, while the levels of the NS1 protein did not change (Fig 5E). In vivo, treatment with 5Z significantly reduced RORγ, HMGCR, nSREBP2, and phosphorylated TAK1 levels in the lungs of H5N1-infected mice (Fig 5F). However, it did not significantly lower the levels of the NP protein (Fig 5F), which is likely due to some compensatory effects such as inhibition of cell death and maintenance of intercellular junction integrity that may increase IAV replication [39–41]. Nevertheless, these results suggest that IAV activates TAK1 to induce RORγ and HMGCR expression, promoting cholesterol biosynthesis and enhancing virus replication.

**Fig 6:**
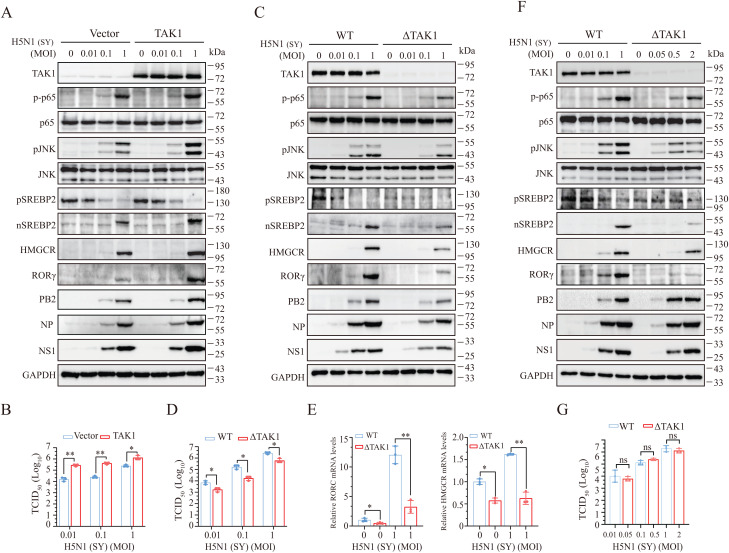
TAK1 activation induces RORγ expression to enhance cholesterol biosynthesis. (A and B) NL20 cells were transfected with the empty vector or the vector encoding TAK1. After incubation for 48 h, the cells were infected with the indicated MOIs of H5N1 and incubated for 16 h. Cell lysates were analyzed for the levels of the indicated proteins (A). Conditioned media were collected and analyzed for virus titers by measuring the TCID_50_ assay (B). Data represents the mean ± SD of three independent experiments. *p < 0.05, **p < 0.01. (C and D) Control and TAK1-deficient NL20 cells infected with the indicated MOIs of H5N1 (SY) were incubated for 16 h. Cell lysates were prepared and analyzed for the expression of cholesterol biosynthesis-related and viral proteins (C). Conditioned medial were collected and analyzed for virus titers by measuring TCID_50_ values (D). △TAK1, TAK1 deficiency. Data are the mean ± SD of three experiments. *p < 0.05, **p < 0.01. (E) Total cellular RNAs from control and TAK1-deficient cells were extracted and analyzed for the levels of *RORC* and *HMGCR* mRNA levels by RT-qPCR. Data represents the mean ± SD of three independent experiments. *p < 0.05, **p < 0.01. (F-G) TAK1-deficient NL20 cells were infected with higher MOIs of H5N1 (SY) than control NL20 cells. After incubation for 16 h, cell lysates were analyzed for the expression of cholesterol biosynthesis-related and viral proteins (F). Conditioned medial were collected and analyzed for virus titers by measuring TCID_50_ values (G). Data are the mean ± SD of three experiments. ns, non-significant.

**TAK1 Activation Upregulates Cholesterol Biosynthesis and Virus Replication by Inducing RORγ Expression**. To confirm the role of TAK1 in regulating RORγ expression, we examined the effects of TAK1 overexpression and knockout on RORγ and HMGCR expression, as well as virus replication. Overexpression of TAK1 modestly enhanced IAV-induced JNK and p65 phosphorylation, RORγ and HMGCR expression, and SREBP2 activation in NL20 cells (Fig 6A). TAK1 overexpression also increased the levels of viral PB2, NP, and NS1 proteins (Fig 6A) and virus titers in the conditioned media of IAV-infected NL20 cells (Fig 6B). Conversely, TAK1 knockout reduced the levels of these viral proteins (Fig 6C) and virus titers (Fig 6D) in the conditioned media of infected NL20 cells. TAK1 knockout also inhibited IAV-induced JNK and p65 phosphorylation, RORγ and HMGCR expression, and SREBP2 activation (Fig 6C). Additionally, TAK1 knockout decreased the levels of RORC and HMGCR mRNAs in IAV-infected NL20 cells (Fig 6E).

To rule out the possibility that the reduced RORγ and HMGCR expression as well as SREBP2 activation resulted from diminished virus replication, we infected TAK1-deficient NL20 cells with higher MOIs of IAV than those used for wild-type cells. Despite the increased viral infection dose, IAV replicated at a comparable rate, as evidenced by similar levels of viral proteins (Fig 6F) and virus titers in the conditioned media (Fig 6G). However, RORγ levels remained much lower in TAK1-deficient cells than in their wild-type controls (Fig 6F). HMGCR expression and SREBP2 activation were also modestly reduced in TAK1-deficient cells (Fig 6F). These observations suggest that TAK1 plays a critical role in regulating IAV infection-induced RORγ expression.

**Fig 7:**
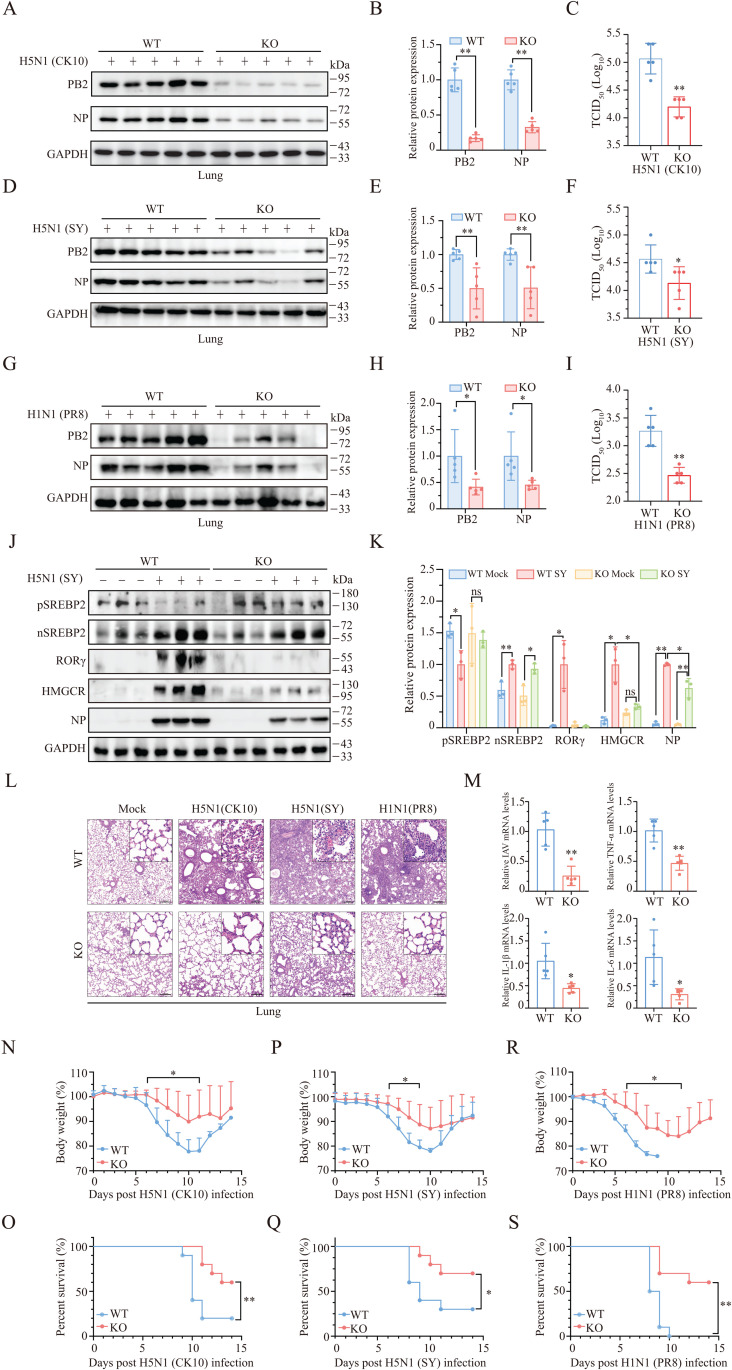
RORγ deficiency inhibits virus replication and prolongs the survival of IAV-infected mice. (A-K) Wild-type and RORγ-deficient male mice (6-8-week-old, 5 mice/group) were infected with two H5N1 strains (CK10 and SY) (5 × 10^4^ pfu/mouse) or H1N1 virus (PR8) (100 pfu/mouse) intranasally. Three days later, mice were sacrificed. One portion of lung tissues were homogenized in RIPA buffer and analyzed for the levels of the indicated proteins by Western blots (**A, D,** G). The band densities from 5 mice per group were analyzed using NIH Image-J software and normalized by the arbitrary units of GAPDH levels. (**B, E,** H). The second portion of lung tissues were homogenized in PBS and analyzed for virus titers (**C, F,** I). Data are the mean ± SD of three experiments. ^*^p < 0.05; ^**^p < 0.01. (J and K) Lung tissue lysates of mock-infected and H5N1 virus (SY)-infected wild-type and RORγ-deficient mice (3 mice/group) were analyzed for SREBP2 activation and the levels of HMGCR and RORγ expression. (L) The sections of paraffin-embedded lung tissues from mice infected with IAV as above were stained with hematoxylin & eosin (H & E). Scale bar, 200 μm. (M) Total RNAs were extracted from one portion of lung tissues from wild-type or RORγ-deficient mice infected with H5N1 virus (SY) and analyzed for the mRNA levels of the viral M1 gene and inflammatory cytokines. Data represents the mean ± SD of the lung tissues from 5 animals per group. *p < 0.05, **p < 0.01. (N-S) Male wild-ty*p*e and RORγ-deficient C57BL/6 mice (6-8-week-old, 10 mice/grou*p*) were infected intranasally with two H5N1 strains, CK10 (**N and O**) and SY (P and Q) (5 × 10^4^ pfu/mouse), or with H1N1 virus (PR8) (R and S) (100 pfu/mouse). Mice were weighed daily and monitored for survival for 14 days. Percent bodyweight changes (**N, P, R**) and percent survival (**O, Q, S**) were plotted. *p < 0.05, **p < 0.01, com*p*ared to the wild-ty*p*e controls.

**RORγ-Deficient Mice Resist IAV Infection.** To investigate the role of RORγ in facilitating IAV replication, we assessed whether RORγ-deficient mice were resistant to IAV infection. The levels of viral proteins and virus titers in lung tissues were significantly lower in RORγ-deficient mice infected with two H5N1 strains (CK10 & SY) and the H1N1 virus (PR8) than in their wild-type counterparts (Fig 7A-I). IAV infection significantly increased SREBP2 activation, as well as RORγ and HMGCR expression in the lungs compared to uninfected controls (Fig 7J and 7K). However, in RORγ-deficient mice, IAV infection poorly induced HMGCR expression and SREBP2 activation compared to wild-type mice infected with H5N1 virus (SY) (Fig 7J and 7K). RORγ deficiency also significantly reduced inflammatory cell infiltration in the lungs of mice infected with both H5N1 (CK10 & SY) and H1N1 (PR8) viruses (Fig 7L). Consistent with this, viral mRNA levels, along with TNF-α, IL-1β, and IL-6 mRNA levels, were significantly decreased in the lungs of RORγ-deficient mice infected with H5N1 virus (SY) (Fig 7M). Furthermore, the mean body weight and survival of RORγ-deficient mice infected with H5N1 or H1N1 viruses were significantly higher than those of wild-type controls infected with the same strains (Fig 7N-S). These findings collectively suggest that RORγ plays a crucial role in facilitating IAV replication.

**Fig 8:**
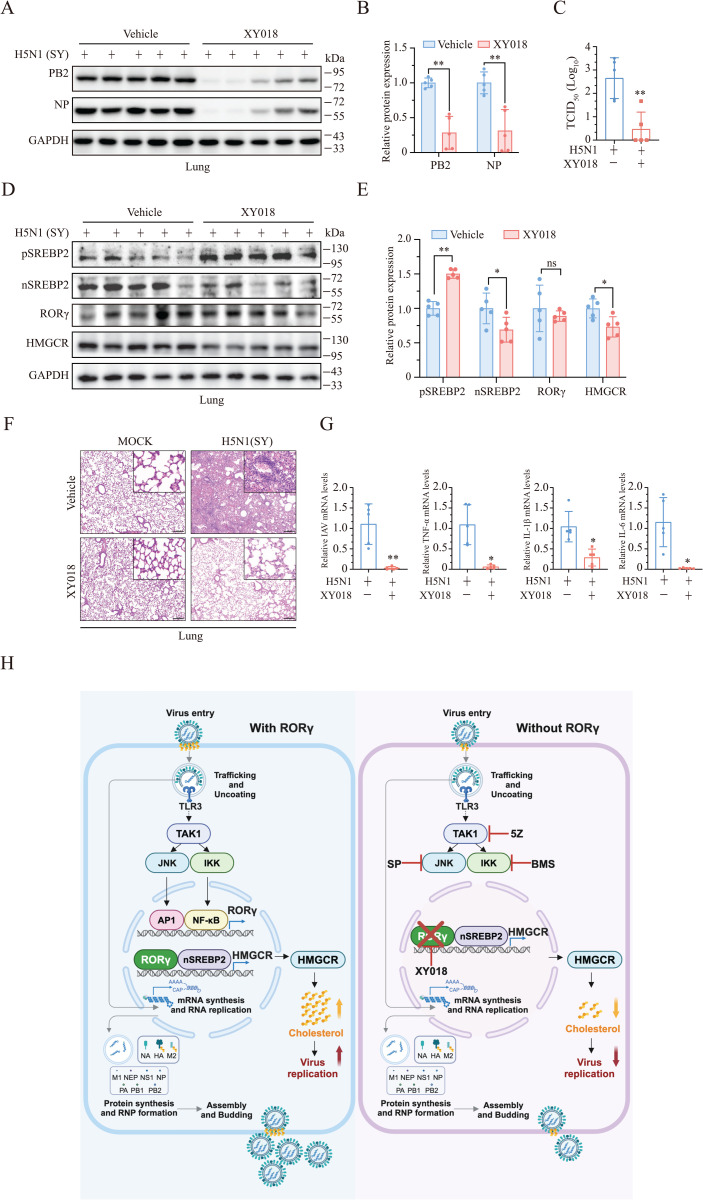
XY018 treatment inhibits IAV replication in vivo. (A-E) Male C57BL/6 mice (6-8-week-old, 5 mice/group) were intranasally pretreated with XY018 (5 mg/kg). Twelve hours later, mice were mock-infected or infected with H5N1 virus (5 × 10^4^ pfu/mouse) and treated daily with the vehicle or XY018 (5 mg/kg body weight) for 3 days. One portion of lung tissues were homogenized in RIPA lysis buffer. Tissue lysates were analyzed for the levels of the viral proteins (**A**) and cholesterol biosynthesis-related proteins (**D**) by Western blot. The density of the bands was analyzed by using NIH Image-J software and normalized by the arbitrary units of GAPDH (B and E). ns, non-significant, ^*^p < 0.05, ^**^p < 0.01. The second portion of lung tissues were homogenized in PBS and analyzed for virus replication by measuring TCID_50_ values (C). Data are the mean ± SD of three experiments. ^**^p < 0.01. The third portion of lungs were fixed in 4% paraformaldehyde. The sections of paraffin-embedded tissue blocks were stained with H & E and visualized for the infiltration of inflammatory cells. Scale bar, 200 μm (F). Another portion of the lungs were homogenized in TRIZOl, total RNAs were extracted and analyzed for the levels of viral and inflammatory cytokine mRNAs (G). Data are the mean ± SD of three experiments. ^*^p < 0.05, ^**^p < 0.01. (H) The schematic model of cholesterol biosynthesis regulation by IAV infection. The viral RNA of IAV binds TLR3 to activate TAK1 and induce RORγ ex*p*ression by AP1 and NF-κB, which are activated by JNK and IKK, respectively. RORγ cooperates with SREBP2 to induce HMGCR expression to promote cholesterol biosynthesis and virus replication. Inhibition of cholesterol biosynthesis by the RORγ inhibitor XY018 leads to the suppression of virus replication. The TAK1 inhibitor 5Z and the inhibitors of JNK and NF-κB can all inhibit RORγ expression, cholesterol biosynthesis, and virus replication.

**XY018 Treatment Inhibits IAV Replication In Vivo.** Finally, we tested whether inhibition of RORγ activity by XY018 could inhibit IAV replication in vivo. XY018 treatment significantly reduced the levels of viral PB2 and NP proteins in the lung tissues of treated mice, compared to untreated controls on day 3 post-infection (Fig 8A and 8B). Virus titers were significantly lower in the lung tissues of XY018-treated mice than in untreated controls on day 3 post-infection (Fig 8C). XY018 treatment also significantly inhibited SREBP2 activation and lowered the levels of HMGCR expression (Fig 8D and 8E). Furthermore, XY018 treatment reduced inflammatory cell infiltration (Fig 8F) and decreased viral mRNA levels, as well as TNF-α, IL-1β, and IL-6 mRNA levels in the lung tissues of H5N1 virus-infected mice on day 3 after infection (Fig 8G). These observations suggest that XY018 inhibits virus replication in vivo by inhibiting RORγ expression.

## Discussion

Cellular cholesterol plays a critical role in viral binding, endocytosis, trafficking, assembly, and budding [42]. We recently demonstrated that IAV infection activates STAT3 to induce SREBP2 expression, thereby enhancing cholesterol biosynthesis [28]. In this study, we present multiple lines of evidence that RORγ is a key transcription factor that plays an even more significant role in regulating cholesterol biosynthesis and IAV replication: 1) IAV infection induces RORγ and HMGCR expression in a dose- and time-dependent manner; 2) RORγ deficiency and specific inhibition by XY018 and GSK805 reduce both cholesterol biosynthesis and IAV replication; 3) RORγ overexpression enhances HMGCR expression and increases virus replication; 4) Supplementation with exogenous cholesterol reverses the inhibitory effect of XY018 on IAV replication; 5) Both XY018 and RORγ deficiency inhibit IAV replication in vivo, with RORγ knockout also prolonging survival in IAV-infected mice. Mechanistically, we show that activation of TAK1 and its downstream kinases, IKK and JNK, which activate NF-κB and AP1, respectively, are responsible for IAV-induced RORγ expression (Fig 8H). Inhibition of TAK1, IKK, and JNK, as well as TAK1 deficiency, all block the expression of cholesterol biosynthesis-related genes and suppress IAV replication (Fig 8H). These findings provide critical mechanistic insights into how IAV infection regulates cholesterol biosynthesis.

Emerging evidence suggests that virus infections increase cholesterol biosynthesis to support replication [14,43]. For example, SREBP2 activation is elevated in monocytes from COVID-19 patients, and its inhibitor, Fatostatin, suppresses the expression of pro-inflammatory cytokines such as TNF-α, IL-1β, and IL-6 [44,45]. Clinical investigations also reveal that elevated HMGCR levels in the blood increase susceptibility to COVID-19 and the risk of hospitalization [46]. The EBNA2 protein of Epstein-Barr virus directly interacts with the HMGCR promoter and cooperates with SREBP2 to induce HMGCR expression, while Dengue virus inhibits HMGCR phosphorylation via AMPK inhibition, activating HMGCR to promote cholesterol synthesis [47,48]. RORγ has also been identified as a key regulator of cholesterol metabolism in tumors [25,49]. In vitro and in vivo studies in prostate and triple-negative breast cancer models demonstrate that RORγ knockdown and antagonists inhibit the expression of cholesterol biosynthesis genes [25,49]. Additionally, liver-specific RORγ deletion significantly reduces liver and serum cholesterol levels in mice on a high-fat diet (HFD). In the present study, we demonstrate that IAV infection induces RORγ and HMGCR expression. RORγ knockout inhibited both HMGCR expression and IAV replication in vitro and in vivo. Collectively, these observations suggest that IAV infection upregulates cholesterol biosynthesis through RORγ expression.

TAK1, a serine/threonine kinase activated by a wide range of cytokine receptors and innate immune signaling pathways, plays a pivotal role in regulating inflammation and antiviral responses through NF-κB activation [50,51]. Interestingly, recent studies highlight TAK1’s involvement in lipid metabolism regulation. TAK1 knockout impairs liver fat uptake and triglyceride synthesis in mice, leading to reduced plasma triglyceride and cholesterol levels when fed a high-fat diet [52]. Furthermore, alcohol consumption activates the TAK1-AMPK axis, promoting cholesterol biosynthesis [53].. TAK1 inhibition has also shown promise in preventing non-alcoholic fatty liver disease [54–57]. In IAV infection, viral RNA binds TLR3 and activates TAK1 [58,59]. Our study shows that TAK1 deficiency and the TAK1 inhibitor 5Z suppress RORγ and HMGCR expression in IAV-infected cells, even when virus replication remains unaffected. These findings suggest that TAK1 activation enhances cholesterol production, largely by inducing RORγ expression.

To further elucidate the mechanisms by which TAK1 regulates RORγ expression, we examined the roles of NF-κB and AP1, key transcription factors in regulating inflammatory cytokine expression and Th17 cell differentiation [60–63]. The p65 subunit of NF-κB and the JunB subunit of AP1 heterodimerize with the basic leucine zipper ATF-like transcription factor (BATF) to bind the RORγ promoter and drive RORγ expression [60,61,64]. Our study demonstrated that inhibition of NF-κB by the IKK inhibitor BMS-345541 and inhibition of AP1 by the JNK inhibitor SP600125 suppressed RORγ and HMGCR expression, even when IAV replication was not inhibited (Fig 5B-E). Moreover, the combined inhibition of both NF-κB and AP1 synergistically suppressed RORγ and HMGCR expression (Fig 5E). Consistent with previous studies, both NF-κB and AP1 have been shown to promote IAV replication and are potential targets for antiviral therapy [65–69]. Based on our findings, we propose that TAK1-induced activation of NF-κB and AP1 promotes IAV replication, at least in part, by inducing RORγ expression and enhancing cholesterol biosynthesis (Fig 8H). However, it is important to note that these inhibitors may have off-target effects that influence virus replication, and additional studies are needed to fully elucidate the role of JNK and NF-κB in IAV replication. For example, JNK may enhance virus replication by promoting autophagy [29,30], while NF-κB may facilitate replication through the induction of Bcl-2 and inhibition of apoptosis.

Interestingly, although TAK1 inhibition reduced cholesterol biosynthesis and virus replication in vitro, as well as RORγ and HMGCR levels in the lungs of IAV-infected mice, it did not significantly decrease viral protein levels (Fig 5F). This observation aligns with our previous studies showing that 5Z treatment does not inhibit virus replication in vivo, nor does it prolong survival in IAV-infected mice [39,41]. This complexity likely arises from TAK1’s broad roles in cell death, autophagy, and inflammation, which may influence both viral replication and lung inflammation [29,39,41,70,71]. Thus, TAK1 inhibition has intricate effects on IAV replication and host responses in vivo.

IAV, as an enveloped RNA virus, relies on cholesterol-rich membranes for replication. The viral envelope is derived from the host cell membrane, with cholesterol constituting approximately 42% of the total lipid content of the virion [10,42,72]. During infection, IAV depletes cellular nutrients, including lipids, to meet the demands of rapid replication [73,74]. Cholesterol depletion from the cell membrane or the IAV envelope, via methyl-β-cyclodextrin, significantly impairs viral infectivity [31,75]. Furthermore, the HA and M2 proteins of IAV are cholesterol-modified to facilitate trafficking and budding [34,76–79]. Given these critical roles, anti-cholesterol agents have gained attention as potential antiviral therapies. Statins, commonly prescribed cholesterol-lowering drugs, have been shown to reduce mortality in patients with pneumonia [17,18]. Atorvastatin blocks IAV-induced lipid droplet formation and inhibits virus replication [80], while Gemfibrozil administration in H2N2 virus-infected mice prolongs survival [81]. However, traditional HMGCR inhibitors may induce compensatory upregulation of HMGCR expression, limiting their ability to effectively reduce cellular cholesterol and inhibit virus replication [10]. Our findings show that while atorvastatin induces HMGCR and SQLE expression in uninfected cells (S3A Fig), XY018 not only inhibits these cholesterol biosynthesis genes but also blocks atorvastatin-induced expression (S3A Fig). Moreover, XY018 combined with 25-hydroxylcholesterol (25-HC) synergistically suppresses the expression of cholesterol biosynthesis genes and enhances antiviral effects (S3B Fig).

SREBP2 is a central regulator of cholesterol biosynthesis [10,11]. Li et al. [82] recently reported that the SREBP2 inhibitor Fatostatin blocks lipid droplets and inhibits IAV replication in vitro and in vivo. However, SREBP2 inhibitors have significant toxicity, limiting their clinical use [10,83]. RORγ inhibitors, such as XY018, are currently being investigated as novel anti-inflammatory agents [22]. In our study, both XY018 and Fatostatin effectively suppressed cholesterol biosynthesis-related genes in uninfected cells (S4A and S4B Fig). However, RORγ overexpression had a greater impact on enhancing IAV replication compared to SREBP2 overexpression (S4C and S4D Fig). Furthermore, XY018 combined with Fatostatin showed a synergistic effect in suppressing IAV replication (S4E and S4F Fig). The combined use of XY018 or Fatostatin with conventional cholesterol-lowering drugs can enhance antiviral activity at lower doses, reducing side effects in vivo.

We are aware that our current work has a couple of limitations. First, whether the RORγ inhibitor or RORγ deficiency inhibits IAV replication in vivo by affecting Th17 cell differentiation and function remains to be defined. RORγt, a variant of RORγ expressed specifically in Th17 cells [84], plays a distinct role in immune regulation, particularly in autoimmune diseases and inflammatory responses. In contrast, RORγ is widely expressed across tissues and affects various biological processes beyond immunity, including lipid metabolism, bone formation, and circadian rhythm regulation [22]. Recent studies suggest that RORγ also contributes to the expression of inflammasome-related genes, which can promote inflammatory responses [85]. Our study shows that XY018 and RORγ deficiency both inhibit the production of inflammatory cytokines, suggesting that targeting RORγ could mitigate both virus replication and inflammation. Further investigation is needed to determine the impact of RORγ inhibitors on IL-17 production, Th17 cell differentiation, and their therapeutic efficacy in IAV-infected mice. Second, whether the RORγ inhibitor or RORγ deficiency also affects virus fusion, endocytosis, trafficking, assembly, and budding remains unclear. Goronzy et al. [86] reported that cholesterol enhances the binding avidity of influenza virus by promoting the small-scale clustering of glycosphingolipid receptors. These authors recently reported that cholesterol depletion in the plasma membrane enhances the fusion of virions [87]. Tang et al. [32] reported that sphingomyelin (SM)-sequestered cholesterol, but not accessible cholesterol, is essential for the clathrin-mediated endocytosis of IAV. It appears that cholesterol plays a complex role in IAV docking, fusion, and endocytosis. Our preliminary investigation suggests that RORγ inhibitors inhibit virus replication in part by blocking virus docking. Whether RORγ impacts other processes during virus replication needs further investigation.

In conclusion, our study demonstrates that IAV infection induces RORγ expression and activates SREBP2 to enhance cholesterol biosynthesis and facilitate virus replication in respiratory epithelial cells and in vivo. Mechanistically, TAK1 activation of NF-κB and AP1 promotes RORγ expression, thereby enhancing cholesterol biosynthesis and IAV replication. Targeting RORγ or its upstream regulators, such as NF-κB and AP1, offers a potential strategy to inhibit cholesterol biosynthesis and reduce IAV replication. Our findings reveal a novel role for RORγ in virus replication and suggest that targeting RORγ, in combination with other cholesterol biosynthesis regulators like HMGCR or SREBP2, may provide a promising approach to control IAV infections.

## Materials and methods

### Ethics statement

Use of animals was approved by the Institutional Animal Care and Use Committee of Yangzhou University and carried out in accordance with the Guide for the Care and Use of Laboratory Animals by the National Research Council.

### Reagents and antibodies

XY018 was synthesized by WuXi AppTec (Shanghai, China). GSK805 (Cat No. S6767), Atorvastatin (Cat No. S5715) and Fatostatin (Cat No. S8284) were purchased from Selleck Chemicals (Houston, TX, USA). 25-Hydroxycholesterol (25-HC) (Cat No. 5741) was purchased from R & D Systems (Minneapolis, MN, USA). Water-soluble cholesterol (Cat No. C4951), 5Z-7-oxozeaenol (Cat No. O9890) and Dimethyl sulfoxide (DMSO) (Cat No. D2650) were purchased from Sigma-Aldrich (Saint Louis, MO, USA). SP600125 (Cat No. 8177) was purchased from Cell Signaling Technology (Danvers, MA, USA). BMS-345541 (Cat No. HY-10519) was purchased from MedChemExpress (Monmouth Junction, NJ, USA). Filipin III (Cat No. 480-49-9) was purchased from Cayman Chemical (Ann Arbor, MI, USA). Sytox Green nucleic acid stain (Cat No. S7020) and TurboFect Transfection Reagent (Cat No. R0532) were purchased from Thermo Fisher Scientific (Waltham, MA, USA). RNA isolater Total RNA Extraction Reagent (Cat No. R401-01), HiScript II 1st Strand cDNA Synthesis Kit (+gDNA wiper) (Cat No. R212-02) and ChamQ Blue Universal SYBR qPCR Master Mix (Cat No. Q312-02) were purchased from Vazyme (Nanjing, China). 2 × EasyTaq PCR SuperMix (+dye) (Cat No. AS111–11) was purchased from TransGen Biotech (Nanjing, China). BCA Protein Assay Kit (Cat No. P0012) was purchased from Beyotime Biotech Inc (Shanghai, China). CellTiter-Glo Luminescent Cell Viability Assay (Cat No. 7572) was purchased from Promega (Madison, WI, USA). Mouse monoclonal anti-RORγ (Cat No. MAB6109) and mouse monoclonal anti-SREBP2 (Cat No. MAB7119) were purchased from R & D Systems (Minneapolis, MN, USA). Rat monoclonal anti-RORγ (Cat No. 14-6988-82) was purchased from Thermo Fisher Scientific (Waltham, MA, USA). Rabbit monoclonal anti-SREBP2 (Cat No. ab30682) was purchased from Abcam Limited (Cambridge, UK). Mouse monoclonal anti-HMGCR (Cat No. sc-271595), Mouse monoclonal anti-NS1(Cat No. sc-130568), Mouse monoclonal anti-HMGCS (Cat No. sc-373681), Mouse monoclonal anti-SQLE (Cat No. sc-271651), Mouse monoclonal anti-FDFT1 (Cat No. sc-271602), Mouse monoclonal anti- mevalonate; diphosphate decarboxylase (MVD) (Cat No. sc-376975), Mouse monoclonal anti- mevalonate kinase (MVK) (Cat No. sc-390669), Mouse monoclonal anti-β-actin (Cat No. sc-8432), Mouse monoclonal anti-glyceraldehyde 3-phosphate dehydrogenase (GAPDH) (Cat No. sc-47724) were purchased from Santa Cruz (Dallas, TX, USA). Rabbit monoclonal anti-Phospho-NF-κB p65 (Ser536) (Cat No. 3033), Rabbit monoclonal anti-NF-κB p65 (Cat No. 8242), Rabbit monoclonal anti-Phospho-SAPK/JNK (Thr183/Tyr185) (Cat No. 4668), Rabbit monoclonal anti-SAPK/JNK (Cat No. 9252), Rabbit monoclonal anti-Phospho-TAK1 (Thr187) (Cat No. 4536), Rabbit monoclonal anti-TAK1(Cat No. 4505), Anti-rabbit IgG HRP conjugated (Cat No. 7074), Anti-mouse IgG HRP conjugated (Cat No. 7076) and Anti-rat IgG HRP conjugated (Cat No. 7077) were purchased from Cell Signaling Technology (Danvers, MA, USA). Rabbit polyclonal anti-PB2 (Cat No. GTX125926) and Rabbit monoclonal anti-NP (Cat No. GTX636247) were purchased from GeneTex (Irvine, CA, USA). Anti-mouse IgG HRP conjugated (Cat No. RA1009–1) was purchased from Vazyme (Nanjing, China). CoraLite594-conjugated Goat Anti-Rabbit IgG(H + L) (Cat No. 7076) was purchased from proteintech (Wuhan, China).

### Cell culture and virus infection

NL20 (an immortalized, nontumorigenic human bronchial epithelial cell line), A549 (a human lung cancer cell line of alveolar epithelial cell origin), MDCK (a Madin-Darby canine kidney cell line), Vero (an African green monkey kidney cell line), and 293T (a human embryonic kidney cell line) cells were obtained from the American Tissue Culture Collection (Manassas, VA, USA). NL20 cells were grown in Ham’s F12 medium with 2.7 g/L glucose, 0.1 mM nonessential amino acids, 0.005 mg/ml insulin, 10 ng/ml epidermal growth factor (EGF), 0.001 mg/ml transferrin, 500 ng/ml hydrocortisone and 4% fetal bovine serum (FBS). A549, MDCK, and 293T cells were grown in DMEM containing 10% FBS. Vero cells were grown in α-MEM with 0.1 mM nonessential amino acids, 1 mM sodium pyruvate, and 10% FBS. LET1 cells, a murine alveolar epithelial type I cell line, were kindly provided by BEI Resources (Manassas, VA). LET1 cells were grown in DMEM containing 10% FBS. MEFs were prepared by trypsin digestion of 13-day embryos of wild-type C57BL/6J mice or RORγ-deficient mice. The 1^st^-3^rd^ passages of monolayers were used for virus infection. All cell lines mentioned above were periodically tested for mycoplasma negative. Experiments with the H5 subtype highly pathogenic avian influenza virus A/mallard/Huadong/S/2005 (H5N1) (SY) and A/chicken/Jiangsu/K0402/2010 (H5N1) (CK10) have been reported previously [28] and were conducted in a BSL-3 level facility. H1N1 virus A/PR/8/1934 virus (PR8) was kindly provided by Dr. Liqian Zhu (College of Veterinary Medicine, Yangzhou University). H1N1 virus A/California/04/09 (CA/09) was kindly provided by Dr. Jinhua Liu (College of Veterinary Medicine, China Agricultural University). Experiments with the H1N1 virus were carried out in the BSL-2 laboratory. IAV stocks were prepared by inoculating 10-day-old specific-pathogen-free embryonic chicken eggs. Virus titers were determined by infecting MDCK cells with 10-fold serially diluted samples (10^1^ to 10^9^). The Reed and Muench method were used to determine the 50% tissue culture infection dose (TCID_50_/100 μl). UV-inactivated H5N1 (dead virus) was generated by placing 600 μl of H5N1 (SY) in a 6-well plate and irradiated under the ultraviolet lamp for 6 h.

### Animals and treatment

C57BL/6 mice (male, 6–8-week-old) were purchased from the Laboratory Animal Center of the College of Veterinary Medicine, Yangzhou University. RORγ KO mice were obtained from the Jackson Laboratory (iBio Logistics, Shanghai, China). All mice were maintained in a specific pathogen-free facility, adhering to a 12-hour light/dark cycle.

To examine the role of RORγ in facilitating IAV replication, wild-type and RORγ-deficient male mice (6–8-week-old) (5 mice/group) were infected with two H5N1 strains (CK10 and SY) (5 × 10^4^ pfu/mouse) or H1N1 virus (PR8) (100 pfu/mouse) intranasally. Three days later, mice were sacrificed, and the lung were harvested. One part of the lung tissue was lysed in RIPA lysis buffer (weight/volume, 1:30) and analyzed for the indicated proteins by western blot. A second part of the lung tissue was fixed in 4% paraformaldehyde and embedded in paraffin within 48 h after fixation. The sections of paraffin-embedded blocks were stained with hematoxylin and eosin. A third part of the lung tissue was grinded in Trizol buffer and analyzed for the indicated genes by RT-qPCR. A fourth part of the lung tissue was grinded in PBS, frozen and thawed three times, and used to detect virus titers. Animal survival and clinical disease were monitored for 14 days or until death. Mice were humanely sacrificed by CO2 inhalation when they became moribund or when the loss of body weight decreased by > 25%.

To examine the effect of 5Z treatment on the expression of cholesterol biosynthesis-related genes, mice were first anesthetized by intraperitoneal injection of sodium pentobarbital (100 mg/kg body weight). Mice were then infected with H1N1 virus (1000 pfu/mouse in 25 μL of PBS) by intranasal instillation. The following day, mice were treated daily with either the vehicle or 5Z (2 mg/kg body weight) by intraperitoneal injection for 2 consecutive days. The dosage of 5Z was determined according to our previous study in IAV [41]. On the third day post-infection, mice received a final dose of 5Z administered 8 hours before sacrifice. Euthanasia was performed by CO2 inhalation. The lung tissue was lysed in RIPA lysis buffer (at a weight/volume ratio of 1:30) and homogenized. The resulting cell lysates were then subjected to Western blot analysis to detect the proteins of interest.

To evaluate the antiviral effects of XY018 in vivo, C57BL/6 mice (male, 6–8-week-old) were inoculated intranasally with H5N1 virus in (5 × 10^4^ pfu/mouse diluted in PBS (25 μL/mouse). Mice (5/group) were treated at 3 h post-virus infection by intranasal administration with XY018 (5 mg/kg/twice daily) dissolved in the vehicle (5% DMSO, 40% PEG300, 2.5% Tween-80, 52.5% sterile saline) (20 μL/mouse). Control mice were treated with the equal volume of the vehicle. Mice were then treated daily with the same dose of XY018 or vehicle for three days. Mice were humanely sacrificed by CO2 inhalation. One part of the lung tissue was lysed in RIPA lysis buffer (weight/volume: 1:30), homogenized in a tissue homogenizer. Tissue lysates were quantified for protein concentration by BCA Protein Assay Kit and then analyzed for viral proteins and cholesterol biosynthesis-related molecules by Western blot. A second part of the lung tissue was fixed in 4% paraformaldehyde and embedded in paraffin within 48 h after fixation. The sections of paraffin-embedded blocks were stained with hematoxylin and eosin (H & E). A third part of the lung tissue was homogenized in TRIZOL. Total RNA was extracted and analyzed for mRNA levels of viral and inflammatory cytokine genes by RT-qPCR. A fourth part of the lung tissue was homogenized in PBS, frozen, and thawed three times, and used to analyze virus replication by quantifying virus titers.

### Western blot

Cells were harvested and lysed in RIPA lysis buffer (150 mM NaCl, 1% Triton X-100, 0.5% sodium deoxycholate, 0.1% SDS, 50 mM Tris-HCl, pH 8.0 and 1 mM PMSF. Cell lysates were analyzed by Western blot with antibodies against the proteins of interest, followed by horseradish peroxidase-conjugated goat anti-rabbit or anti-mouse IgG and SuperSignal Western Pico enhanced chemiluminescence substrate (Pierce Chemical Co., Rockford, IL). To detect the phosphorylation of JNK, p65 and TAK1, blots were first probed with an antibody against phosphorylated protein. The membrane was then stripped and re-probed with an antibody against their total proteins. Full-length and truncated SREBP2 were detected by probing sliced blots from the same membrane. All Western blot experiments were repeated at least twice with similar results, each with the detection of β-actin or GAPDH as a loading control. The relative phosphorylation levels were analyzed by quantifying the density of the phosphorylated protein bands normalized to their corresponding total proteins. The relative levels of viral proteins were analyzed by quantifying the density of protein bands normalized to the bands of β-actin or GAPDH. The results were presented as bar graphs.

### Real-time quantitative PCR analysis

Total RNA from NL20 cells (Figs 1C, 1D and 4I), wild-type, TAK1-deficient NL20 cells (Fig 6E), or mice lung tissue (Figs 7M and 8G) was extracted by TRIzol reagent. Reverse transcription of RNA was performed using the HiScript III RT SuperMix for qPCR (+gDNA wiper) according to the manufacturer’s protocol. The cDNA was subjected to quantitative real-time PCR using a ChamQ Universal SYBR qPCR Master Mix kit. Amplification of β-actin was included as a control. The sequences of the primers used for RT-qPCR are shown in S1 Table. All Real-Time RT-PCR analyses were performed in triplicate. Results from three independent experiments were pooled and statistically analyzed.

### Virus binding assay

Virus binding assay was conducted as previously reported [88,89]. Briefly, NL20 cells pretreated with XY018 for 8 h were chilled at 4°C for 10 min and then inoculated with 2.5 MOI of SY virus. After incubation at 4°C for 1 h, unbound virions were removed by rinsing three times with cold PBS 3 times. Total RNAs were extracted by directly lysing the cells in TRIzol. Viral mRNA levels of the M gene were quantified by RT-PCR. Results from three independent experiments were pooled and statistically analyzed.

**RORγ, nSREBP2, and TAK1 Transfection.** 293T and NL20 cells were transiently transfected with pLX304-RORγ (kindly provided by Dr. Demin Cai, Yangzhou University), pCAGGS-nSREBP2 [28] and pcDNA3.1-TAK1 (kindly provided by Dr. Xin Lin, Tsinghua University) or their corresponding empty vectors as a control. After incubation for 48 h, the cells were left uninfected or infected with the indicated MOI of the H5N1 virus and then incubated for 16 h. Cell lysates were prepared and analyzed for the proteins of interest. Virus titers in the conditioned media were collected and analyzed for the TCID_50_ values. The results represent the mean ± SD of three independent experiments.

### Gene knockout

sgRNAs were designed by using the Benchling CRISPR Guide Design Software. Oligos corresponding to the sgRNAs were synthesized and cloned into lentiCRISPR v2 vectors. The sgRNA sequences are shown in S1 Table. NL20 and 293T cells seeded in 24-well plates were transfected with the LentiCRISPRv2 vector or the vector encoding sgRNA targeting RORγ, HMGCR, or TAK1. After incubation for 48 h, the monolayers were trypsinized and re-seeded in 6-well plates in the medium containing puromycin (1–2 μg/ml). Fresh media containing the same concentrations of puromycin were changed every three days. After incubation for 14 days, individual colonies were picked, expanded, and analyzed for the expression of RORγ, HMGCR and TAK1 by Western blot. The colonies screened from the cells transfected with a pLenti-V2 empty vector were used as controls. All experiments were carried out with at least two colonies.

### Filipin and immunofluorescence staining

NL20 cells seeded in a 96-well plate in triplicate or on coverslips in 24-well plate were left uninfected or infected with H5N1 (SY) (1 MOI) or H1N1 (PR8) (5 MOI) virus and incubated in the absence or presence of XY018 (2 μM) for 24 hr. The cells were rinsed twice with PBS and then fixed with 4% paraformaldehyde at room temperature for 30 min. After rinsing twice with PBS, the cells were incubated in PBS containing 1.5 mg glycine/ml at room temperature for 10 min to quench paraformaldehyde. Cellular cholesterol was stained with Filipin III, a polyene macrolide antibiotic that binds unesterified cholesterol and produces blue fluorescence light. The cells were stained in Filipin III working solution (50 μg/ml in PBS containing 10% FBS) at room temperature for 2 h. After removal of the Filipin solution and rinse twice with PBS, the cells were blocked at room temperature with 5% bovine serum albumin (BSA) in PBS for 1 hr, and then probed with an anti-NP monoclonal antibody (1:100) in blocking buffer overnight at 4 °C. The cells were then stained with CoraLite594-conjugated Goat Anti-Rabbit IgG (1:500) for 1 hr at room temperature. After removing wash twice with Hank’s balanced salt solution (HBSS), the cells were stained for 15 min in HBSS containing Sytox Green (167 nM), a nuclei acid dye that stain the nucleus with green fluorescence light. The cells were visualized under a Leica confocal microscope (DMI6000 B) or quantified for Filipin (Excitation, 360 nm; Emission, 480 nm), Sytox Green (Excitation, 504 nm; Emission, 523 nm), and CoraLite594 (Excitation 593; Emission 614) fluorescence in a Tecan plate reader (Infinite 200 PRO). The arbitrary units of Filipin and NP signals were normalized with that of Sytox Green. The results represent the mean ± SD of three independent experiments.

**EC**_**50**_ **and CC**_**50**_ **Determination.** NL20 and LET1 cells seeded in a 24-well plate were pretreated with various concentrations of various inhibitors for 8 h. After infection with 0.01 MOI of SY or PR8 virus, the cells were incubated for 24 h in the absence or presence of the same concentrations of these inhibitors. The conditioned media were collected and analyzed for TCID_50_ values. The results from three experiments were pooled and used to calculate the EC_50_ values. The Reed and Muench method [90] was used to determine the 50% tissue culture infection dose (TCID_50_ per 100 ml). The cytotoxicity of GSK805, XY018, and ATV was determined by seeding NL20 or LET1 cells in 96-well plates (3.5 × 10^4^ cells/well) in the presence of various concentrations of these inhibitors for 48 h. Cell viability was measured by using a CellTiter-Glo kit (Promega, Madison, WI, USA). The results from three experiments were pooled and used to calculate the CC_50_ values. Data represents the mean ± SD of three independent experiments.

### Statistical analysis

Differences in mRNA levels, virus titers, Filipin fluorescent intensity, and Western blot band density in the lung tissues from uninfected and IAV-infected mice were statistically analyzed by using an unpaired Student *t* test. Differences in the body weights of wild-type and RORγ-deficient mice were analyzed using a repeated measures ANOVA test. Differences in the survival of wild-type and RORγ-deficient mice were statistically analyzed by using a Log-Rank test. The *p* value of <0.05 was considered statistically significant. All statistics were performed with GraphPad Prism (GraphPad software 8.0.2) (https://www.graphpad.com/scientific-software/prism).

## Supporting information

S1 Fig IAV induces the expression of cholesterol biosynthesis-related proteins in multiple cell lines, supporting Fig 1. (A and B) LET1 and 293T cells were infected with the indicated MOI H5N1 virus for 24 h. Cholesterol biosynthesis-related proteins were detected by Western Blot. (C) NL20 cells were infected with H5N1 or UV-inactivated H5N1 virus at an indicated MOI for 24 h. Cholesterol biosynthesis-related proteins were detected by Western Blot.
(TIF)

S2 Fig RORγ promotes IAV replication in NL20 cells, supporting Fig 3. (A) Control and RORγ knockdown NL20 cells were infected with H5N1 at the indicated MOI for 16 h. Cell lysates were prepared and analyzed for cholesterol biosynthesis-related proteins and viral proteins levels. (B) NL20 cells were transfected with the empty vector or the vector encoding RORγ. After incubation for 48 h, the cells were infected with H5N1 for an additional 16 h. Cell lysates were prepared and analyzed for cholesterol biosynthesis-related proteins and viral proteins levels.
(TIF)

S3 Fig XY018 blocks ATV-induced HMGCS and SQLE expression. (A and B) NL20 cells seeded in a 12-well plate were incubated in the absence or presence of the indicated concentrations of ATV or 25-HC minus or plus XY018 (1 μM) for 24 h. (C-F) NL20 cells pretreated with the indicated concentrations of ATV or 25-HC minus or plus XY018 (0.5 μM) for 8 h were infected with 0.01 MOI H5N1 (SY) virus and then incubated for 24 h in the presence of the same concentrations of ATV or 25-HC minus or plus XY018. Untreated control cells were treated with 0.1% dimethyl sulfoxide (DMSO). Cell lysates were prepared and analyzed for the levels of indicated proteins by Western blot (C and E). β-actin was detected as a loading control. Conditioned medial were collected for measuring TCID50 values (D and F). The results represent one of three independent experiments with similar results. Data are the mean ± SD of three experiments. ns, non-significant; *p < 0.05, **p < 0.01.
(TIF)

S4 Fig RORγ and SREBP2 synergistically enhance viral replication. (A and B) NL20 cells seeded in a 12-well plate were incubated in the absence or presence of the indicated concentrations of XY018 (A) or fatostatin minus or plus XY018 (1 μM) (B) for 16 and 24 h. (C and D) SREBP2 cooperates with RORγ to enhance IAV replication. NL20 cells were transiently transfected with the empty expression vector or the vector encoding nSREBP2 or RORγ. After incubation for 48h, the cells were infected with 0.1 MOI H5N1 for another 16 h. Cell lysates were prepared and analyzed for the expression of viral proteins by Western blots (C). The conditional media were collected and analyzed for virus titers by measuring the TCID50 values (D). (E and F) NL20 cells pretreated with the indicated concentrations of fatostatin minus or plus XY018 (0.5 μM) for 8 h were infected with 0.01 MOI H5N1 (SY) virus and then incubated for 24 h in the presence of the same concentrations of fatostatin minus or plus XY018. Untreated control cells were treated with 0.1% dimethyl sulfoxide (DMSO). Cell lysates were prepared and analyzed for the levels of indicated proteins by Western blot (E). β-actin was detected as a loading control. Conditioned medial were collected for measuring TCID50 values (F). The results represent one of three independent experiments with similar results. Data are the mean ± SD of three experiments. ns, non-significant; *p < 0.05, **p < 0.01.
(TIF)

S5 Fig XY018 and fatostatin synergistically inhibit the expression of cholesterol biosynthesis-related genes. (A-C) NL20 cells seeded in a 12-well plate was first infected with H5N1 virus (SY). After incubation for 8 h, the cells were then incubated in the absence or presence of the indicated concentrations of XY018 (A) and fatostatin (B) alone or in combination (C) for 16 h. Cell lysates were prepared and analyzed for the expression of cholesterol biosynthesis-related genes by Western blots with their specific antibodies.
(TIF)

S1 Table Primers used for RT-PCR and gene knockout. (DOCX)
