# Supplementary figures and images for: Influenza virus infection reprograms cholesterol biosynthesis to facilitate virus replication by the TAK1-RORγ axis
Source: PLoS Pathog. 2025 Oct 24;21(10):e1013646. doi: 10.1371/journal.ppat.1013646 (PMC12574845; doi:10.1371/journal.ppat.1013646)

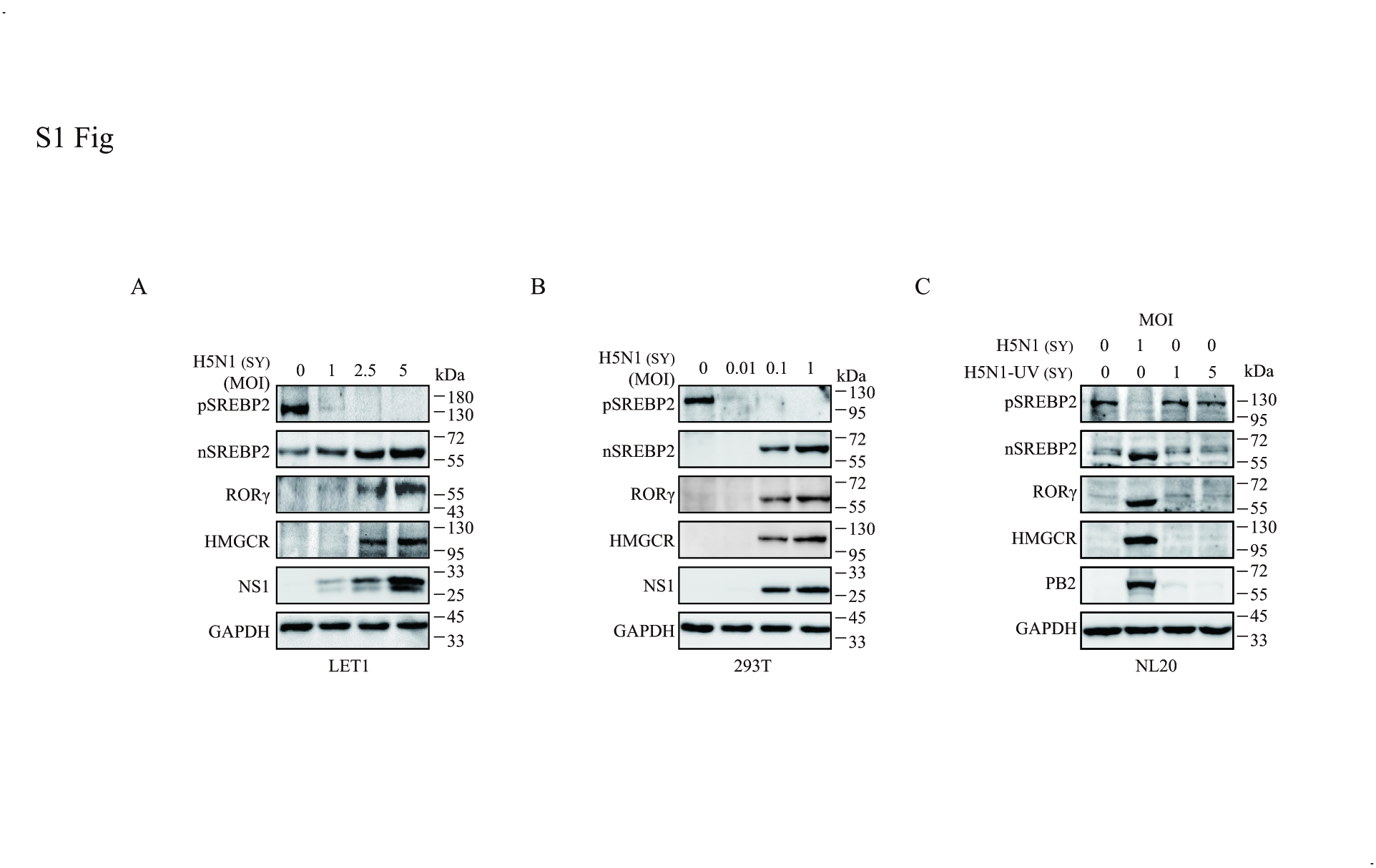

Supplement: S1 Fig — Cholesterol biosynthesis-related proteins were detected by Western Blot. (C) NL20 cells were infected with H5N1 or UV-inactivated H5N1 virus at an indicated MOI for 24 h. Cholesterol biosynthesis-related proteins were detected by Western Blot. (TIF) [file ppat.1013646.s001.tif]

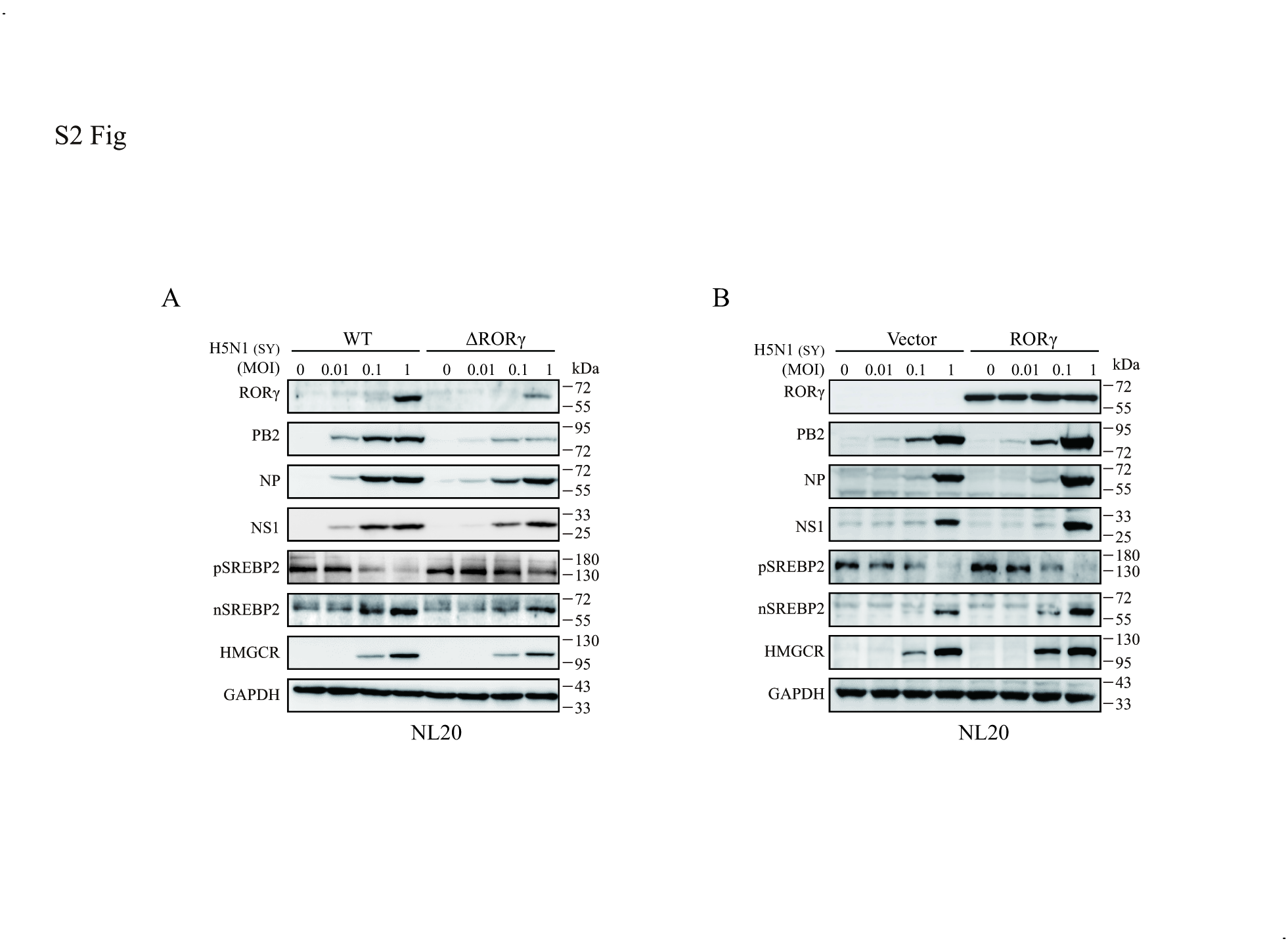

Supplement: S2 Fig — Cell lysates were prepared and analyzed for cholesterol biosynthesis-related proteins and viral proteins levels. (B) NL20 cells were transfected with the empty vector or the vector encoding RORγ. After incubation for 48 h, the cells were infected with H5N1 for an additional 16 h. Cell lysates were prepared and analyzed for cholesterol biosynthesis-related proteins and viral proteins levels. (TIF) [file ppat.1013646.s002.tif]

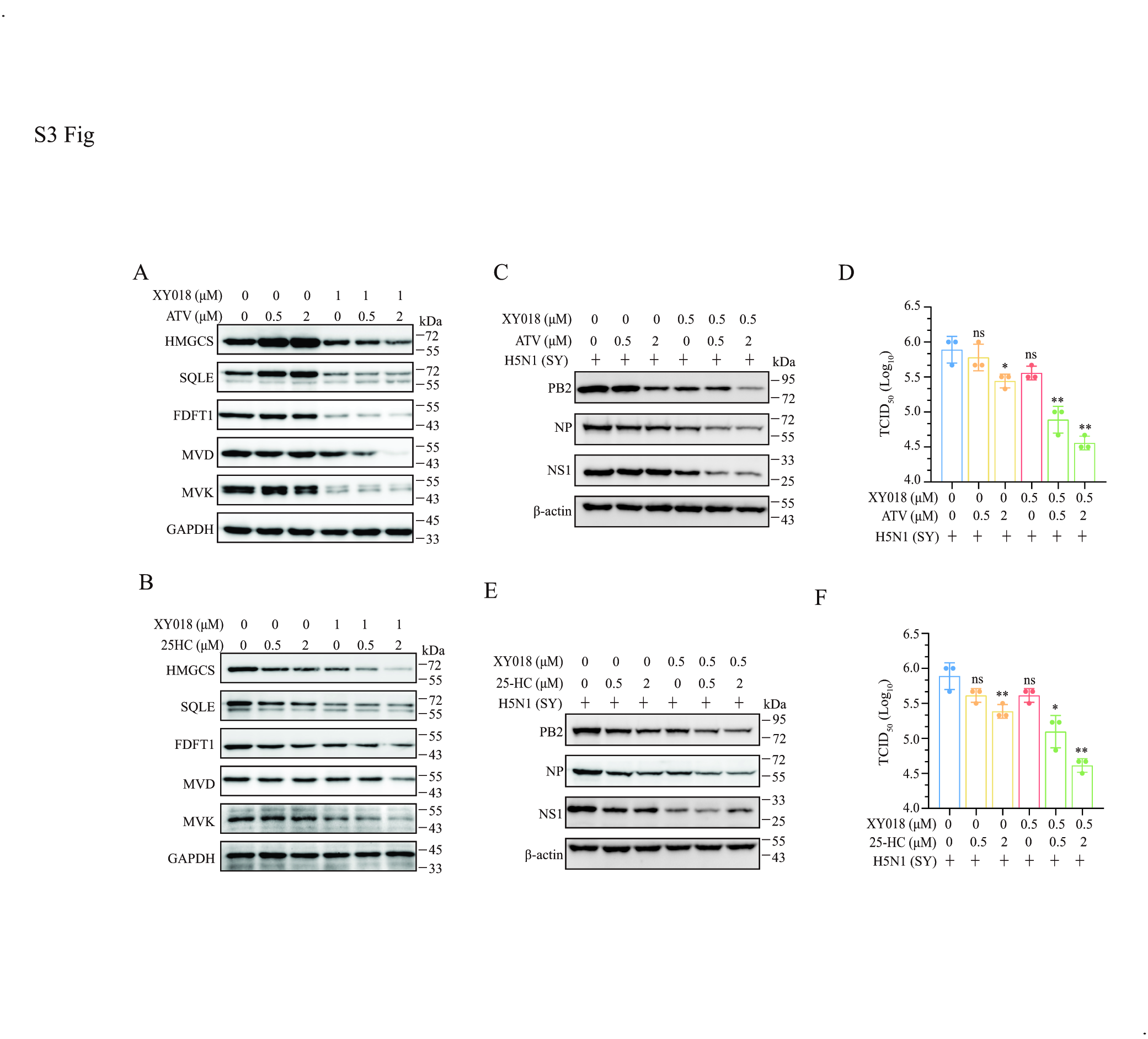

Supplement: S3 Fig — (A and B) NL20 cells seeded in a 12-well plate were incubated in the absence or presence of the indicated concentrations of ATV or 25-HC minus or plus XY018 (1 μM) for 24 h. (C-F) NL20 cells pretreated with the indicated concentrations of ATV or 25-HC minus or plus XY018 (0.5 μM) for 8 h were infected with 0.01 MOI H5N1 (SY) virus and then incubated for 24 h in the presence of the same concentrations of ATV or 25-HC minus or plus XY018. Untreated control cells were treated with 0.1% dimethyl sulfoxide (DMSO). Cell lysates were prepared and analyzed for the levels of indicated proteins by Western blot (C and E). β-actin was detected as a loading control. Conditioned medial were collected for measuring TCID50 values (D and F). The results represent one of three independent experiments with similar results. Data are the mean ± SD of three experiments. ns, non-significant; *p < 0.05, **p < 0.01. (TIF) [file ppat.1013646.s003.tif]

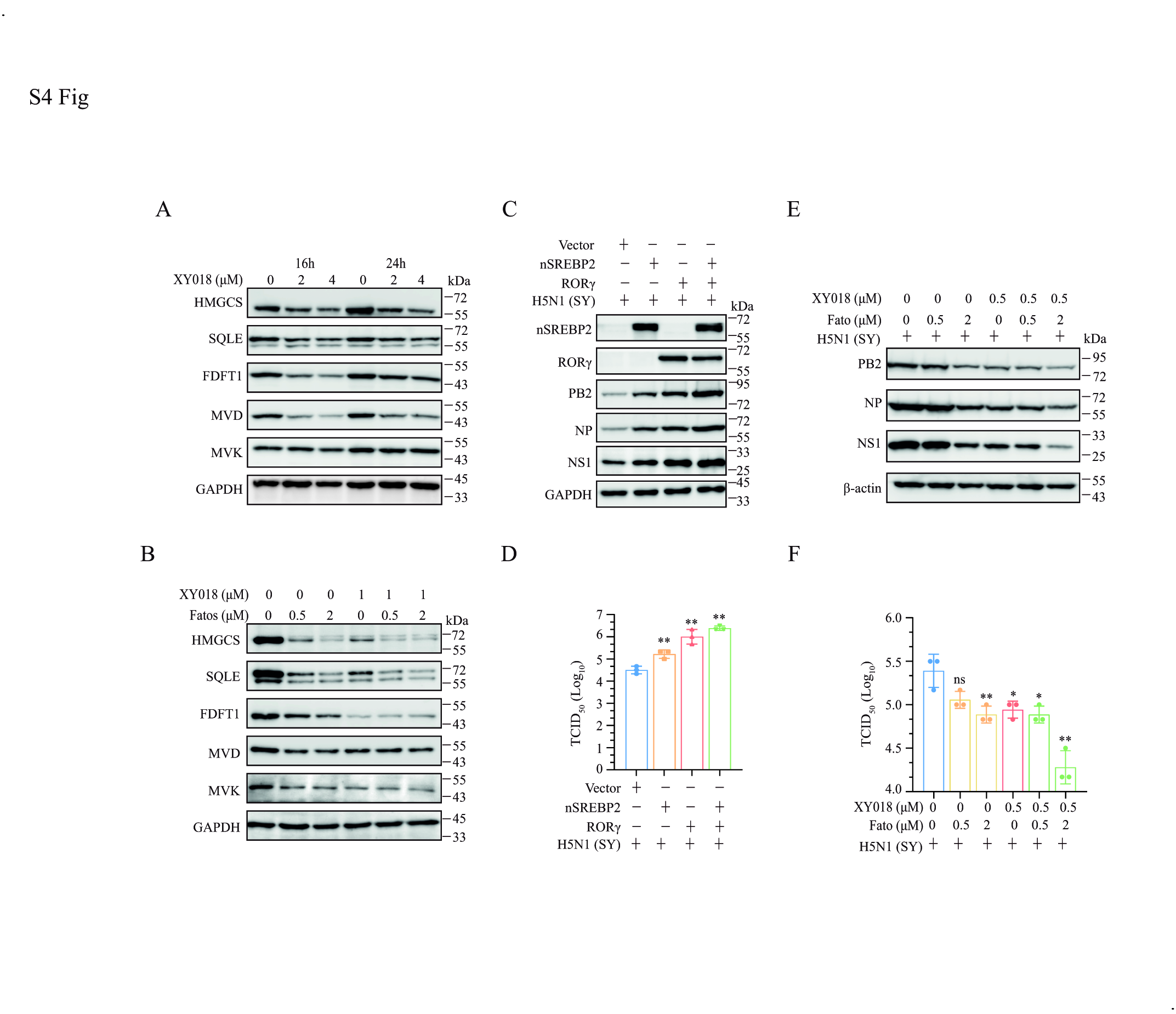

Supplement: S4 Fig — (A and B) NL20 cells seeded in a 12-well plate were incubated in the absence or presence of the indicated concentrations of XY018 (A) or fatostatin minus or plus XY018 (1 μM) (B) for 16 and 24 h. (C and D) SREBP2 cooperates with RORγ to enhance IAV replication. NL20 cells were transiently transfected with the empty expression vector or the vector encoding nSREBP2 or RORγ. After incubation for 48h, the cells were infected with 0.1 MOI H5N1 for another 16 h. Cell lysates were prepared and analyzed for the expression of viral proteins by Western blots (C). The conditional media were collected and analyzed for virus titers by measuring the TCID50 values (D). (E and F) NL20 cells pretreated with the indicated concentrations of fatostatin minus or plus XY018 (0.5 μM) for 8 h were infected with 0.01 MOI H5N1 (SY) virus and then incubated for 24 h in the presence of the same concentrations of fatostatin minus or plus XY018. Untreated control cells were treated with 0.1% dimethyl sulfoxide (DMSO). Cell lysates were prepared and analyzed for the levels of indicated proteins by Western blot (E). β-actin was detected as a loading control. Conditioned medial were collected for measuring TCID50 values (F). The results represent one of three independent experiments with similar results. Data are the mean ± SD of three experiments. ns, non-significant; *p < 0.05, **p < 0.01. (TIF) [file ppat.1013646.s004.tif]

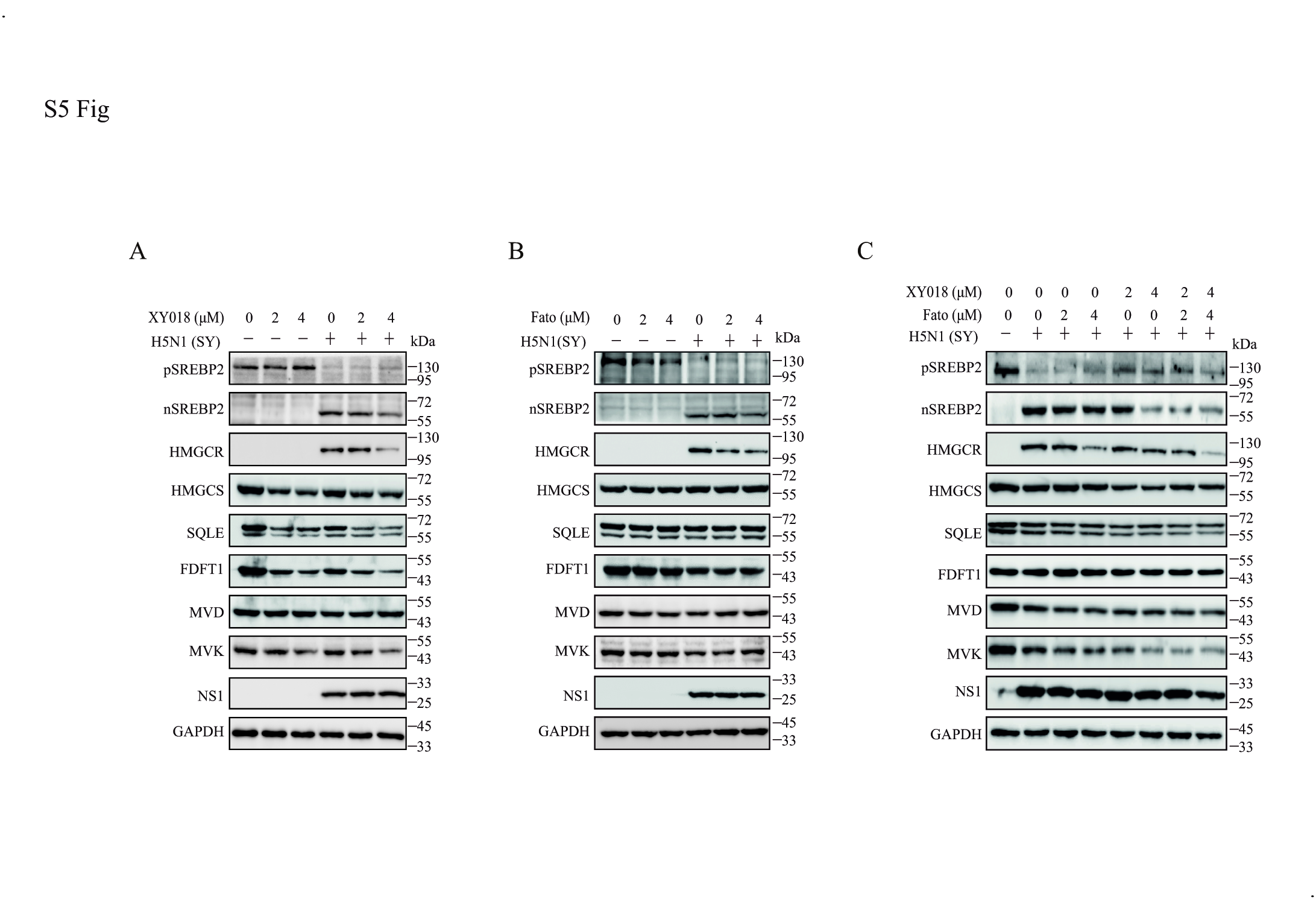

Supplement: S5 Fig — (A-C) NL20 cells seeded in a 12-well plate was first infected with H5N1 virus (SY). After incubation for 8 h, the cells were then incubated in the absence or presence of the indicated concentrations of XY018 (A) and fatostatin (B) alone or in combination (C) for 16 h. Cell lysates were prepared and analyzed for the expression of cholesterol biosynthesis-related genes by Western blots with their specific antibodies. (TIF) [file ppat.1013646.s005.tif]
