# Supplementary material for: Influenza virus infection reprograms cholesterol biosynthesis to facilitate virus replication by the TAK1-RORγ axis
Source: PLoS Pathog. 2025 Oct 24;21(10):e1013646. doi: 10.1371/journal.ppat.1013646 (PMC12574845; doi:10.1371/journal.ppat.1013646)
Supplement: S1 Table — (DOCX) [file ppat.1013646.s006.docx]

| Primers for RT-qPCR | |
| --- | --- |
| Human_RORC_rtF | AGATACCCTCACCTACACCTTG |
| Human_RORC_rtR | CCGCTCAGGGCTGTATTCAA |
| Human_HMGCR_rtF | CCCAGCCTACAAGTTGGAAA |
| Human_HMGCR_rtR | GCTCCCATCACCAAGGAGTA |
| Human_SREBP2_rtF | AACGGTCATTCACCCAGGTC |
| Human_SREBP2_rtR | GGCTGAAGAATAGGAGTTGCC |
| Human_ACTB_rtF | CACCATTGGCAATGAGCGGTTC |
| Human_ACTB_rtR | AGGTCTTTGCGGATGTCCACGT |
| Mouse_Il-1β_rtF | TGGACCTTCCAGGATGAGGACA |
| Mouse_Il-1β_rtR | GTTCATCTCGGAGCCTGTAGTG |
| Mouse_Tnf-α_rtF | CCCTCACACTCAGATCATCTTCT |
| Mouse_Tnf-α_rtR | GCTACGACGTGGGCTACAG |
| Mouse_Il-6_rtF | TGAGATCTACTCGGCAAACCTAGTG |
| Mouse_Il-6_rtR | CTTCGTAGAGAACAACATAAGTCAGATACC |
| Mouse_Actb_rtF | CATCCGTAAAGACCTCTATGCCAAC |
| Mouse_Actb_rtR | ATGGAGCCACCGATCCACA |
| IAV Matrix_rtF | GCACTTGATATTGTGGATTCTTGATCGTCTT |
| IAV Matrix_rtR | GACAAAATGACCATCGTCAACATCCACA |
| Primers for sgRNA cloning | |
| gROR_F1 | CACCGGGCAGCCAGGACGGCACCA |
| gROR_R1 | AAACTGGTGCCGTCCTGGCTGCCC |
| gROR_F2 | CACCGAGACAGCACCGAGCCTCAC |
| gROR_R2 | AAACGTGAGGCTCGGTGCTGTCTC |
| gHMGCR_F1 | CACCGTCAAGACTTTTTCGAATGCA |
| gHMGCR_R1 | AAACTGCATTCGAAAAAGTCTTGAC |
| gHMGCR_F2 | CACCGATCCCTGGGAAGTCATAGTG |
| gHMGCR_R2 | AAACCACTATGACTTCCCAGGGATC |
| gTAK1_F1 | CACCGAGTTGTTTGCAAAGCTAAG |
| gTAK1_R1 | AAACCTTAGCTTTGCAAACAACTC |
| gTAK1_F2 | CACCGTAGACCAACAACGAGTCATC |
| gTAK1_R2 | AAACGATGACTCGTTGTTGGTCTAC |

**S1. Table. Primers used for RT-PCR and gene knockout**
